# European Epidemiological Patterns of Cannabis- and Substance-Related Congenital Neurological Anomalies: Geospatiotemporal and Causal Inferential Study

**DOI:** 10.3390/ijerph20010441

**Published:** 2022-12-27

**Authors:** Albert Stuart Reece, Gary Kenneth Hulse

**Affiliations:** 1 Division of Psychiatry, University of Western Australia, Crawley, WA 6009, Australia; 2 School of Medical and Health Sciences, Edith Cowan University, Joondalup, WA 6027, Australia

**Keywords:** cannabis, cannabinoid, mutagenesis, genotoxicity, epigenotoxicity, transgenerational inheritance

## Abstract

Introduction. Of the many congenital anomalies (CAs) recently linked with community cannabis exposure, arguably the most concerning are neurological CAs (NCAs). We therefore conducted a detailed study of this in fourteen European nations. Methods. Congenital anomaly data were from Eurocat. Drug exposure data were from European Monitoring Centre for Drugs and Drug Addiction. Income from World bank. Results. The Netherlands, Spain, France and Bulgaria reported increasing rates of many NCAs. The NCA rate (NCAR) was higher in nations with increasing daily cannabis use when compared to those without (*p* = 0.0204, minimum E-value (mEV) = 1.35). At bivariate analysis, the mEVs of the following NCAs were significantly cannabis related: severe microcephaly 2.14 × 10^13^, craniosynostosis 5.27 × 10^11^, nervous system 4.87 × 10^11^, eye 2.73 × 10^7^, microphthalmos 4.07 × 10^6^, anencephalus 710.37, hydrocephalus 245.64, spina bifida 14.86 and neural tube defects 13.15. At inverse probability, weighted panel regression terms including cannabis were significantly related to the following series of anomalies: nervous system, anencephalus, severe microcephalus, microphthalmos, neural tube defect and spina bifida from *p* = 5.09 × 10^−8^, <2.2 × 10^−16^, <2.2 × 10^−16^, 4.84 × 10^−11^, <2.2 × 10^−16^ and 9.69 × 10^−7^. At geospatial regression, this same series of anomalies had terms including cannabis significant from *p* = 0.0027, 1.53 × 10^−7^, 3.65 × 10^−6^, 2.13 × 10^−8^, 0.0002 and 9.76 × 10^−12^. 88.0% of 50 E-value estimates and 72.0% of mEVs > 9. This analysis therefore demonstrates both close association of cannabis exposure with multiple NCAs across space-time and also fulfills the quantitative criteria of causal inferential analysis. Conclusions. Nine NCARs on bivariate and six NCARs on multivariable regression were cannabis related and fulfilled quantitative epidemiological criteria for causality and are consistent with other series. Particular concerns relate to exponential dose–response effects demonstrated in the laboratory and epidemiological studies. Great caution with community cannabinoid penetration is warranted. Data indicate that cannabis is a significant environmental teratogen and thus imply that cannabinoids should be regulated similarly to the manner in which all other important genotoxins are carefully controlled by communities for their self-sustaining longevity and the protection of generations yet to come.

## 1. Introduction

With increasing recent reports describing many congenital anomalies (CAs) attributable to prenatal cannabis exposure, one of the most concerning groups of anomalies is that affecting the brain and neurological development generally [1,2]. With classical studies in the preclinical literature describing hydrocephalus, encephalocele, spina bifida and anencephalus in rats, rabbits and hamsters gestationally exposed to cannabis [3,4,5], and recent confirmatory epidemiological findings relating to encephalocele and hydrocephaly [6] and CDC reports of anencephalus [7], reports of neural tube elevations in Canada [8], increased hydrocephalus in Australia [9] and several increased rates of encephalocele, spina bifida and microphthalmia in the USA [10,11], we were concerned about the profile that would be encountered in Europe where some nations such as Spain, France, Portugal, the Netherlands have reported increasing rates of cannabis use [12,13].

Many of these anomalies affected or were known to relate to brain development. Since congenital causes of being intellectually challenged can be severe, can remain throughout life and may often impose severe degrees of disability, this is an intrinsically severe and important group of congenital anomalies to understand. Moreover, since prenatal cannabis exposure had previously been linked with anencephalus [6,7], autism [14,15,16] and smaller head sizes [17,18,19], we enquired if this apparent continuum of increasing brain damage would be continued in the European dataset, particularly as that database includes an anomaly called “severe microcephalus” which is not tracked elsewhere.

Both cannabinoid genotoxicity [20,21,22,23,24,25,26,27,28,29,30] and cannabinoid-induced disruptions of mitochondrial activity [31,32,33,34,35,36], which support and supply key substrates and energy to genomic and epigenomic reactions [37], have been extensively documented to demonstrate exponential cannabis dose–response relationships. Moreover, these exponential effects [22,23,24,25,26,30,35] have been confirmed in several epidemiological studies which document a substantial discontinuous step in congenital anomaly rates from the fourth to the highest quintile of cannabis exposure [1,10,11]. Since many parts of Europe are well documented to have seen abrupt rises in community exposure to cannabinoids resulting from increased prevalence of cannabis use, cannabis use daily intensity, and increased Δ9-tetrahydrocannabinol (THC) potency in marketed cannabis products [12,13], it seemed likely that this would transition the community from the low risk genotoxic zone into the level at high risk for major and serious genotoxic outcomes. Indeed, there is some powerful evidence that this may indeed be occurring via contamination of the food chain at least in those parts of northeastern France where cannabis crops are grown and both calves and human babies are being born limbless [38,39,40].

We decided to include some eye anomalies in this group as the eye is formed as the confluence of two sets of tissues: one coming from the face, which is responsible for the formation of the anterior segments of the eye including the lens, and an outgrowth of the forebrain and optic nerve which is responsible for the posterior parts of the eye including the retina and neural elements [41]. For this reason, we treat anterior eye anomalies separately under a companion paper addressing orofacial disorders. However, the decision was made to include whole-of-eye anomalies in this section. The data report both anophthalmos and anophthalmos/microphthalmos. These two disorders behave quite differently analytically. It seems to us that the former condition referred to anophthalmos properly so-called and the latter actually refers to microphthalmos. These disorders have therefore been treated this way in the following analysis.

For all of these reasons, we were keen to study central nervous system anomalies in the European dataset both in terms of the bivariate associations and also in terms of their multivariable modelling within causal inferential and space-time paradigms. The following report sets out this detailed account. The specific hypotheses investigated were as follows: (1) Did previously described bivariate relationships between cannabis exposure and neurological CAs (NCAs) persist after multivariate adjustment; (2) Did these relationships persist when considered formally in a space-time context; and (3) Did these relationships fulfill the formal criteria of quantitative causal inferential analysis.

## 2. Methods

### 2.1. Data

Data on all available congenital anomaly rates were downloaded for each of 14 nations by each individual year from the European Network of Population-Based Registries for the Epidemiological Surveillance of Congenital Anomalies (EUROCAT) website [42] and analyzed. It is important to note that EUROCAT total congenital anomaly rate includes anomaly rates amongst live births, stillbirths and cases where early termination for anomaly was practiced all combined together so that it represents a total overall picture across all classes of births. The nations selected were chosen on the basis of the availability all or most of their congenital anomaly data across the years 2010–2019. National alcohol (liters of pure alcohol consumed per capita annually) and tobacco (percent daily tobacco use prevalence) use data were downloaded from the World Health Organization [43]. Drug use data for amphetamines cannabis and cocaine were taken from the European Monitoring Centre for Drugs and Drug Addiction (EMCDDA) [44]. Last month cannabis use data were also supplemented by data on the tetrahydrocannabinol (THC) content of cannabis herb and resin provided in recent reports which have been published [13]. Data on daily cannabis use are also available from EMCDDA and was consistent with data collated in recent reports [13]. Median household income data (in USD) were taken from the World Bank [45].

### 2.2. National Assignment

Nations were categorized as being either high and/or rising daily cannabis use or low and/or falling daily cannabis use based on a recent European epidemiological study of cannabis use in Europe (see Supplementary Figure S4 in reference [13]). Thus, Italy, the Netherlands, Norway, France, Germany, Belgium, Croatia, Portugal and Spain were categorized as nations experiencing increasing daily use, while Hungary, Poland, Finland, Bulgaria, and Sweden were nations which were experiencing low or falling levels of daily cannabis use.

### 2.3. Derived Data

Because several metrics of cannabis use, exposure and consumption were available, it was possible to calculate various derived compound metrics. For this reason, last month cannabis use prevalence data were multiplied by the THC content of cannabis herb and resin to derive their products. These metrics were then multiplied by imputed daily cannabis use rates to derive further compound metrics for both cannabis herb and resin.

### 2.4. Data Imputation

Linear interpolation was used to complete missing data. This was particularly used for daily cannabis use. In all, 59 data points on daily cannabis use from EMCDDA were available for these 14 nations in this period. Linear interpolation was used to expand this dataset to 129 datapoints (further details provided in Results section). Swedish data on cannabis resin THC concentration were not available. However, it was noted that the resin/herb THC concentration was virtually constant in nearby Norway at 17.7 so this ratio was multiplied by the Swedish cannabis herb THC concentration data to derive estimates of Swedish cannabis resin THC concentration. Similarly, Polish data for the cannabis resin THC concentration were unavailable. The resin to herb THC concentration ratio of nearby Germany was used to estimate the resin THC content in Poland from the known herb THC concentrations observed in Poland. Since geospatial analytical techniques do not tolerate missing data, the techniques of last observation carried forward or backwards for Croatia in 2018 and 2019 and the Netherlands in 2010 were used to complete missing data. Multiple imputation methods could not be applied to this dataset as it is not possible to apply multiple imputed datasets in panel or spatial multivariable regression techniques.

### 2.5. Statistics

R Studio version 1.4.1717, based on R version 4.1.1 from the Comprehensive R Archive Network and the R Foundation for Statistical Computing [46], was used for data processing. The analysis was conducted in December 2021. Dplyr from the tidyverse was used for data manipulation [47]. Log transformation of datasets was employed as appropriate to improve compliance with normality assumptions based on the results of the Shapiro–Wilks test. ggplot2 from the tidyverse was used to draw graphs. Maps were drawn using ggplot2 together with sf (simple features) [48] and custom color palettes and palettes taken from the viridis and viridisLite packages were used to generate fill schemas [49]. The R package colorplaner was used to draw and color bivariate maps [50]. Illustrations have not been published previously and are original. Linear regression was performed from Base R. The package nlme was used for mixed effects regression [51]. The classical technique for model reduction, namely serial deletion of the least significant term, was used in all multivariable models to yield a final reduced model which has been model presented. Multiple linear models were processed in a single pass using combined techniques from R packages broom and purrr [47,52,53]. The overall effect of covariates in multivariable models may be quantified and this is denoted as the “marginal effect”. In this study, the overall marginal effect in multivariate models was calculated using the R package margins [54].

### 2.6. Covariate Selection

The presence of so many different metrics for cannabis exposure and consumption created a major problem for analysis as it was not clear which was the most appropriate metric to employ in any particular analytical scenario. Indiscriminate use of excessive covariates in a multivariable model would make models impossible to analyze by unnecessarily consuming degrees of freedom and thereby restrict ability to assess interactions. This issue was formally addressed by employing random forest regression using the R package ranger [55] with variable importance being formally studied using the R package vip (variable importance plot) [56]. The most highly predictive set of covariates from this process was entered into the regression modelling equations. The Results section presents the tables from this analysis.

### 2.7. Panel and Geospatial Analysis

R package plm [57] was used to conduct panel analysis across both space and time simultaneously using the “twoways” effect. The sparse spatial weights matrix was calculated using R package spdep (spatial dependency) [58] by using the edge and corner “queen” relationships. The spatial panel random effects maximum likelihood (spreml) function from the package spml, which allows detailed modelling and correction of model error structures [59,60] was used for geospatial modelling. Such models may produce four model coefficients of interest and these in turn are useful in determining the most appropriate error structure for the model. These coefficients are rho the spatial coefficient, phi the random error effect, psi the serial correlation effect, and theta the spatial autocorrelation coefficient. In each case, the most appropriate error structure was chosen for each spatial model generally, taking care to preserve the model error specification across closely related models. The appropriate error structure was determined by the backwards methods from the full general model to the most specific model, as has been previously described [61]. Temporal lagging by one or two years, as indicated, was applied to both panel and geospatial models.

### 2.8. Causal Inference

The formal tools of causal inference were used for this analysis. Inverse probability weighting (ipw) is the technique of choice to convert a purely observational study into a pseudo-randomised study and from these models it is entirely appropriate to draw causal inferences [62]. All multivariable panel models presented in the present study were inverse probability weighted. The R package ipw was used for inverse probability weighting. Similarly E-values (expected values) is a powerful form of sensitivity analysis and may be used to quantify the correlation required of some hypothetical unmeasured confounder covariate with both the exposure of concern and the outcome of interest in order to explain away some apparently causal relationship [63,64,65]. It therefore provides a quantitative measure of the robustness of the model to extraneous covariates which have not been accounted for within the parameters which have been selected and measured. E-Values are associated with a corresponding confidence interval and the 95% lower bound of this confidence interval is reported herein as the minimum E-Value confidence interval. E-Value estimates greater than 1.25 have been reported to indicate causality [66] and E-values greater than nine have been described as high [67]. The R package EValue [68] was used for the calculation of E-values. Both E-values and inverse probability weighting are foundational and pivotal techniques used in formal causal inferential methods in order to allow causal relationships to be assessed from pseudorandomized real world observational studies.

### 2.9. Data Availability

Raw datasets including 3800 lines of computation code in R has been made freely available through the Mendeley data repository at the following URLs: 10.17632/vd6mt5r5jm.1 and 10.17632/gr5ntsbp7p.1.

### 2.10. Ethics

Ethical approval for this study was provided from the Human Research Ethics Committee of the University of Western Australia number RA/4/20/4724 on 24 September 2021.

## 3. Results

### 3.1. Input Data

Supplementary Table S1 sets out the baseline data for this study. It shows the 14 nations which contributed data via the Eurocat database and the 11 congenital anomalies which were studied in this group. As can be seen, there were 1327 datapoints in this dataset with most anomalies having 110 data points each. Drug use data, including several metrics for cannabis exposure including compound indices, are also shown along with median household income.

Eye anomalies were included with disorders of the CNS as the eye is formed embryologically as a confluence of tissues contributed both from the face and the developing forebrain. The facial tissues contribute the anterior segments of the eye and the retina and neural tissues come from the brain. For this reason, congenital anomalies of the front section of the eye are considered in our companion manuscript looking at facial disorders and disorders of the eye as a whole are considered in the present paper.

Daily drug use data were notable incomplete and is shown in Supplementary Table S2. 59 points are listed there. In order to allow these data to be used in analyses, a further 70 datapoints were added by linear interpolation as shown in Supplementary Table S3.

### 3.2. Bivariate Relationships

**Figure 1:**
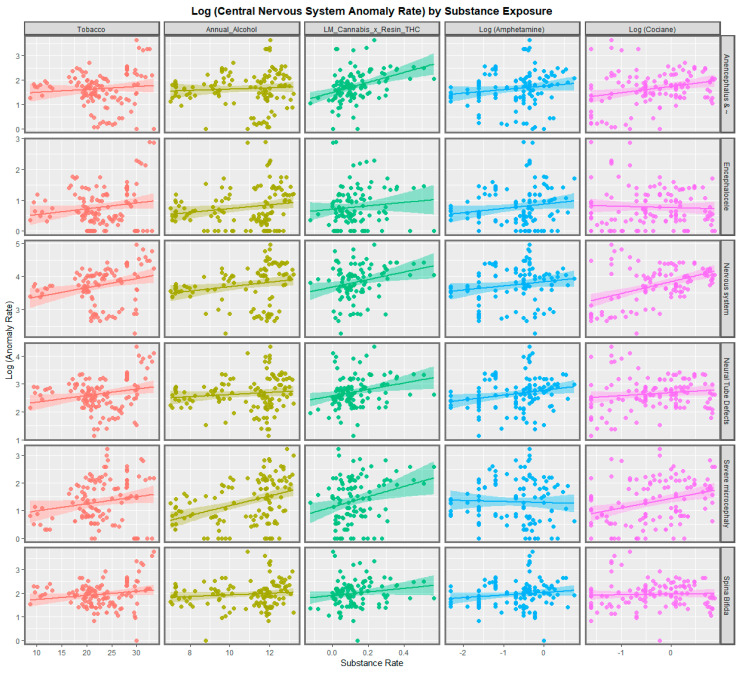
Paneled scatterplots for log (central nervous system congenital anomaly rates) by substance exposure rates for selected anomalies-1.

Figure 1 sets out the bivariate relationship of the various rates of anomalies with the different substance exposures. It is noted that many of the regression line slopes in Figure 1 for tobacco, alcohol amphetamine and cocaine are positive and upward trending. The metric of cannabis use to which they are compared in this Figure is last month cannabis use × cannabis resin THC concentration. The regression lines for this metric in Figure 1 are positive to various degrees.

**Figure 2:**
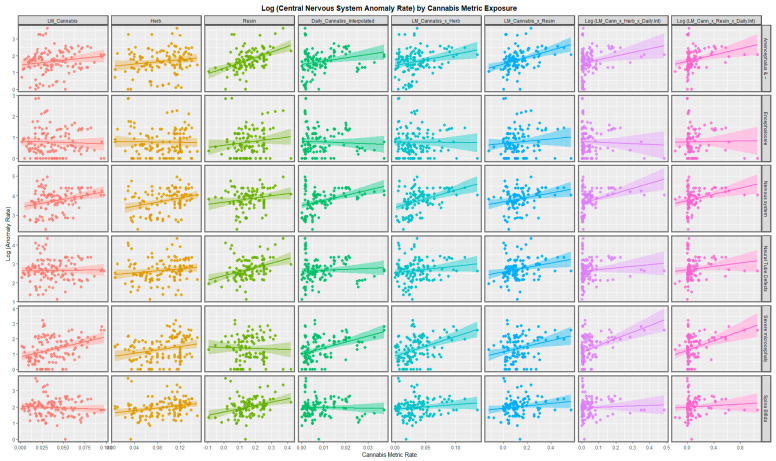
Paneled scatterplots for log (central nervous system congenital anomaly rates) by exposure to various metrics of cannabis for selected anomalies-1.

The regression lines for tobacco, alcohol and amphetamine in Supplementary Figure S1 are non-descript and not significant. For some anomalies, the regression relationships of cocaine are positive and strongly significant. For four of the anomalies listed, the compound cannabis metric used is clearly positive. It is noted in Supplementary Figure S1 that the slopes of the regression lines for the cannabis metric and cocaine are opposite for the two anomalies Anophthalmia/Microphthalmia and anophthalmos. For this reason, these two designations are believed to be reporting on different conditions, the first one is believed to be a surrogate for microphthalmia and the second relates to Anophthalmia properly so-called. Scatterplots for six anomalies are shown in Figure 2 and for five NCAs in Supplementary Figure S2.

Figure 2 and Supplementary Figure S2 illustrate the relation of the different anomalies to the various cannabis exposure metrics. Particularly strong and positive relationships are noted for cannabis resin THC concentration and also daily cannabis use interpolated and the various combinations of these indices. Other relationships are as shown.

**Figure 3:**
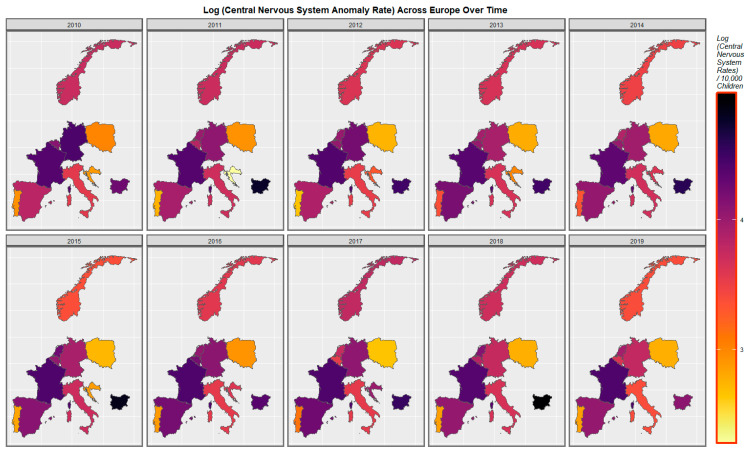
Sequential map-graphs of log (central nervous system anomaly rates) across surveyed European nations over time, 2010–2019.

Figure 3 is a graphical map showing the rate of central nervous system conditions across Europe over the decade 2010–2019. The area of France appears to have constantly relatively high rates. The rate in Germany started high but has declined from there. The rate has increased across the decade in Spain.

**Figure 4:**
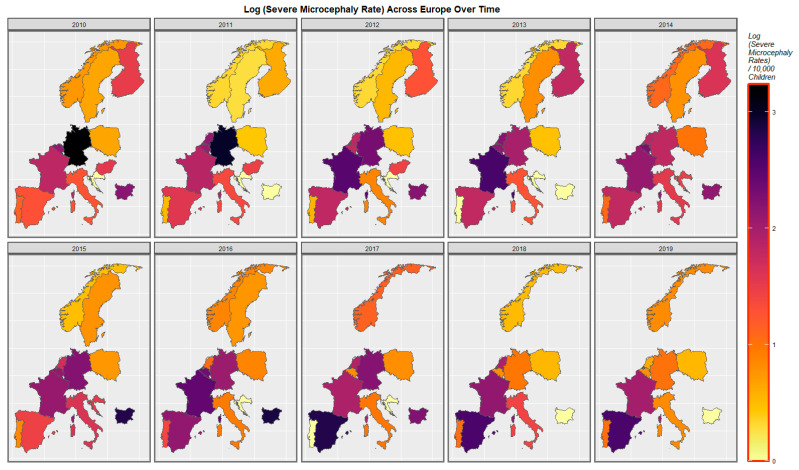
Sequential map-graphs of log (severe microcephaly rates) across surveyed European nations over time, 2010–2019.

Figure 4 shows the pattern of severe microcephaly across the decade. For this anomaly, the situation in Spain and France has declined whilst that in Germany and Belgium has improved. Rates in the Netherlands have fluctuated.

**Figure 5:**
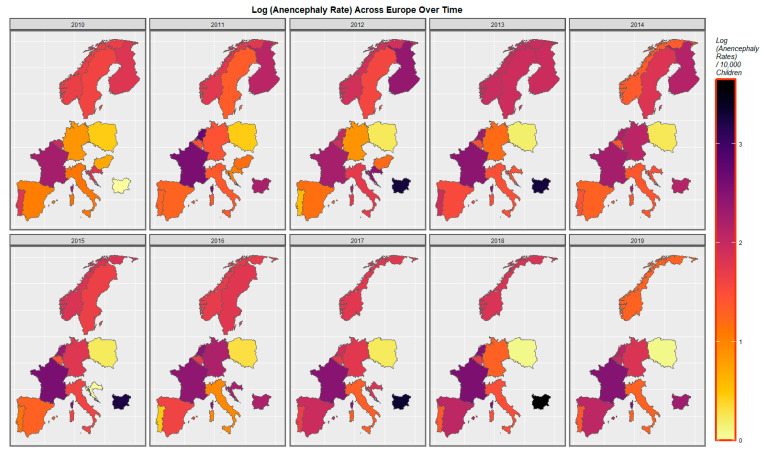
Sequential map-graphs of log (anencephalus rates) across surveyed European nations over time, 2010–2019.

The space-time patterns of anencephaly are as shown in Figure 5. Rates in Spain, France, Germany and Belgium have increased. Bulgaria has often had a high rate. Polish rates have been relatively lower and have declined across the decade. Rates in the low countries have fluctuated across a relatively high range.

The pattern of microphthalmia is shown in Supplementary Figure S3. For the reasons described above, it is felt that these data reflect microphthalmia more than anophthalmia which appear to be different entities. Rates in Poland have been persistently low and have declined. Those elsewhere have fluctuated.

Rates of neural tube defects (Supplementary Figure S4) have been persistently low in Poland but have deteriorated in France, Germany and Spain. They have often been relatively high in Bulgaria.

Eye anomalies (Supplementary Figure S5) were persistently high in Finland when data were available and rates have increased in France. German and Italian rates have declined across this period.

Rates of one of the compound cannabis metrics, last month cannabis use: cannabis resin THC are shown in Supplementary Figure S6. This index is noted to have increased in Spain, the Netherlands, France and Portugal across this period.

**Figure 6:**
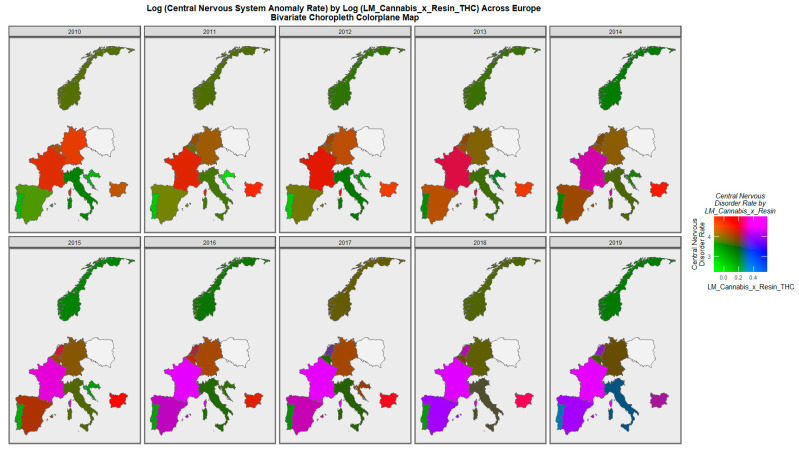
Bivariate colorplaner sequential map-graphs of log (central nervous system anomaly rates) by log of last month cannabis use: cannabis resin THC concentration across surveyed European nations over time, 2010–2019.

Figure 6 is a bivariate plot of overall central nervous system anomaly rates compared to the compound cannabis exposure metric last month cannabis use: cannabis resin THC concentration. One reads the graph by noting that the areas which are high for both covariates are shaded purple or pink. Thus, the maps clearly illustrate the emergence of simultaneously high rates of both parameters in France, Spain, Bulgaria and the Netherlands across the decade.

**Figure 7:**
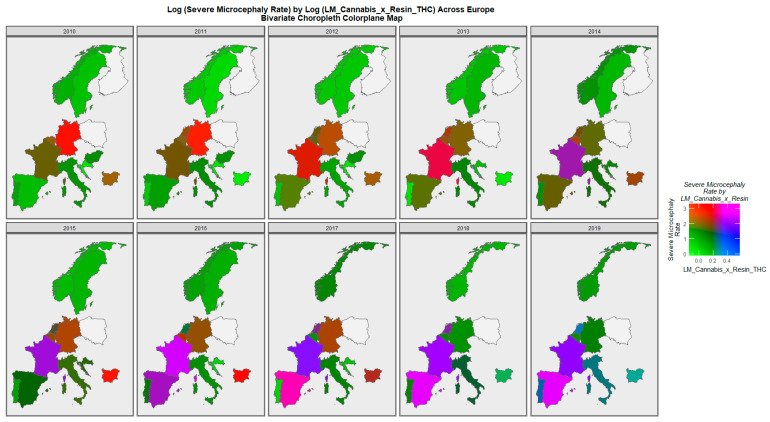
Bivariate colorplaner sequential map-graphs of log (severe microcephaly rates) by log of last month cannabis use: cannabis resin THC concentration across surveyed European nations over time, 2010–2019.

The pattern of severe microcephaly plotted against the same compound cannabis exposure parameter is shown in Figure 7. Again, dually high rates of both parameters are noted to have emerged in France, Spain and the Netherlands across this decade.

When microphthalmos is considered, the areas of France and Spain are noted to have turned shades of purple across this period, which again indicates dually concordant elevated rates (Supplementary Figure S7).

The convergent pattern of neural tube defects across the continent is shown in Supplementary Figure S8 where simultaneously elevated rates are noted to have occurred in France, the Netherlands and Bulgaria. The rate in Germany is noted to have risen but without a rise in this particular metric of cannabis exposure.

### 3.3. Comparing High and Low Cannabis Use Countries

As described in the Methods section, this group of nations may be separated into those where daily cannabis use is increasing and those where it is decreasing or stable. When nations are dichotomized in this way, the appearance for all the anomalies considered together is as shown in Supplementary Figure S9. At linear regression, it is found that the rate in the nations where cannabis use is higher is significantly greater than those where it is not (β-est. = 0.155, t = 2.332, *p* = 0.0265; model Adj.R.Squ. = 0.0029, F = 4.934, *p* = 0.0265). When this issue is considered by each anomaly, the appearance shown in Supplementary Figure S10 is seen. In this Figure, it is clear that the rates in the two sets of nations overlap when considered overall, a finding confirmed by mixed effects regression. However, in the severe microcephaly group there is a significant difference between the groups (β-est. = 0.372, t = 2.349, *p* = 0.0205; model Adj.R.Squ. = 0.0360, F = 5.517, *p* = 0.0205).

Supplementary Table S4 shows details from 132 linear regression models for each anomaly considered against each of the substance exposure metrics. From these models, those with positive regression coefficients and significant *p*-values were extracted as shown in Supplementary Table S5. Sixty models were thus selected. They are listed in descending order of their minimum E-values. Importantly, it is noted that daily cannabis use interpolated and severe microcephaly head up this list of bivariate associations. Forty-seven of these 60 (78.3%) substance exposure terms include cannabis compared to only seven (11.7%) for cocaine, two (3.3%) for tobacco and only one (1.7%) for alcohol.

### 3.4. Multivariable Analysis

The next step is to move from the bivariate context to multivariable regression. However, it is unclear which selection of the 13 exposure covariates is most suitable for each CA as the set of independent variables. Random Forrest regression was used in tandem with variable importance plots to decide this issue. The six variable importance tables for the six CAs of primary interest are shown as Supplementary Tables S6–S11. The reasons for choosing this set of CAs are described in the Discussion section.

#### 3.4.1. Multivariable Relationships—Panel Regression

Supplementary Table S12 shows a series of inverse probability weighted panel regression models for nervous system disorders as an additive and an interactive model and a model lagged by two years. The use of inverse probability weighting is important as it allows us to move from a merely observational study into a pseudo-randomized paradigm from which causal inference can properly be made. One notes in this Table that cannabis exposure metrics are positive in each model with high levels of statistical significance (from 5.09 × 10^−8^ in the interactive model).

This pattern is repeated across Supplementary Tables S13–S17 which consider, respectively, anencephalus, severe microcephalus, microphthalmos, neural tube defect and spina bifida. These important findings at multivariable regression confirm that in all six cases metrics of cannabis exposure survive model reduction and persist at high levels of statistical significance in final models. This indicates that the strong effects observed at bivariate regression persist after careful adjustment for relevant covariates in additive, interactive and lagged contexts.

#### 3.4.2. Multivariable Relationships—Geotemporospatial Regression

The next issue of relevance was to consider these data in their native space-time context, where important analytical confounding factors such as serial correlation, spatial correlation, random error structures and spatial autocorrelation could be properly and formally accounted for. For this reason, the geospatial links between countries shown in Supplementary Figure S11 were defined, edited and finalized as shown, which then became the basis for the sparse spatial weights matrix entered into the spatial regression equations.

Table 1 presents the final geospatial regression additive, interactive and temporally lagged models along with details of the individual regression parameters employed for nervous system disorders. In the additive and interactive models, terms including cannabis exposure have positive regression coefficients and are statistically significant. This signal is not seen in the temporally lagged models.

Table 2 presents final spatiotemporal regression results for anencephalus. Terms relating to cannabis exposure are not seen in the additive model but are notable with high levels of statistical significance in the interactive and the models lagged to one and two years.

Table 3 shows final geospatial models for severe microcephalus. In this case, cannabis-related terms are seen in each of the models presented and their magnitude dominates the regression result. The effect is not removed by temporal lagging.

Final additive, interactive and lagged geospatial models for microphthalmos are presented in Table 4. Terms with positive regression coefficients are high levels of statistical significance seen in all models.

When neural tube defects are considered in Table 5, cannabis metrics with positive coefficients appear in the additive and both lagged models.

Review of final models for spina bifida confirms this pattern with terms for the cannabis metric being noted in all the geospatial models studied (Table 6).

### 3.5. Causal Inference—E-Values

From each of the regression models, it is possible to extract applicable E-values. The E-value quantitates the expected magnitude of the correlation of some unknown confounding variable with both the exposure of concern and the outcome of interest to explain away an apparently causal effect. It is therefore an important part of quantitative causal inferential techniques.

E-values for panel and geospatial models are presented in Supplementary Tables S18 and S19, respectively. These Tables are then combined and the 50 E-values are shown together in Supplementary Table S20 listed in descending order of minimum E-value. This table is notable for several features. Severe microcephalus and microphthalmos head up the table, as does daily cannabis exposure interpolated. Furthermore, many of the E-values reported are very high, especially when it is noted that values above nine are said to be high [67].

When these 50 E-value estimates are listed out in order as shown in Table 7, 44/50 (88.0%) are in the high range [67] and 50/50 (100%) exceed the threshold of causality [66]. For the minimum E-values (mEV), the applicable Figures are 36/50 (72%) in the high range and 48/50 (96.0%) above the threshold of causality.

Supplementary Table S21 lists these E-values in order of the anomaly to which they apply. These data are then summarized by anomaly in Table 8 where they are listed in descending order of their minimum E-values. Interestingly, all the median minimum E-values are in the high range from 8.79 to 1.04 × 10^29^.

Similarly, these values can be ordered by the regression term involved, grouped into the primary covariate of interest (herb or resin THC content or daily cannabis use, Supplementary Table S22), and summarized (Supplementary Table S23). Groups can be compared using the Wilcoxson test where it is noted that all the inter-group comparisons are statistically significant (Supplementary Table S24). Thus, the order of severity in these multivariable studies is daily cannabis use > herb THC concentration > resin THC concentration.

## 4. Discussion

### 4.1. Main Results

Nine of the eleven NCAs identified were related to metrics of cannabis exposure on bivariate testing and these relationships held through multivariable modelling for all six of the NCAs selected for advanced analysis. These overall results were consistent with other similar recent epidemiological reports in other jurisdictions [6,7,8,9,10,11].

Mapping studies showed that rates of most disorders were relatively severe in France. Rates of most disorders deteriorated in Spain (Figure 3 and Figure 4 and Supplementary Figures S4 and S5). On bivariate maps, graphs with last month cannabis use × resin THC concentration as the independent variable, rates of nervous system disorders deteriorated in the Netherlands, Spain, France and Bulgaria; rates of severe microcephaly deteriorated in the Netherlands, Spain and France; rates of microphthalmos deteriorated in the Netherlands, Bulgaria and France; and rates of neural tube defects deteriorated in the Netherlands, Bulgaria and France (Figure 6 and Figure 7 and Supplementary Figures S7 and S8).

Neurological congenital anomaly rates were higher in nations with increasing daily cannabis use compared to those without increasing daily use (*p* = 0.0204, minimum E-value (mEV) = 1.35; Supplementary Figure S9). Daily cannabis use interpolated had the most significant slopes of all the covariates (Figure 1 and Figure 2, Supplementary Figures S1 and S2, Table 1). NCAs most closely related to cannabis use were severe microcephalus, nervous system disorders, eye disorders and microphthalmos (Table 1).

The order of relationship to cannabis at bivariate analysis was severe microcephaly (mEV = 2.14 × 10^13^) > craniosynostosis (5.27 × 10^11^) > nervous system (4.87 × 10^11^) > eye (2.73 × 10^7^) > microphthalmos > (4.07 × 10^6^) > anencephalus (710.37) > hydrocephalus (245.64) > spina bifida (14.86) > neural tube defects (13.15) (Table 1). After multivariable regression, the order of cannabis relationship by median mEV was severe microcephalus > microphthalmos > nervous system disorders > neural tube defects > anencephalus > spina bifida (Supplementary Table S21).

At inverse probability, weighted panel regression cannabis metric terms were significantly related to the following series of anomalies: nervous system, anencephalus, severe microcephalus, microphthalmos, neural tube defect and spina bifida from *p* = 5.09 × 10^−8^, <2.2 × 10^−16^, <2.2 × 10^−16^, 4.84 × 10^−11^, <2.2 × 10^−16^ and 9.69 × 10^−7^ (Supplementary Tables S12–S17).

At geospatial regression, this same series of anomalies had terms including cannabis significant from *p* = 0.0027, 1.53 × 10^−7^, 3.65 × 10^−6^, 2.13 × 10^−8^, 0.0002 and 9.76 × 10^−12^ (Table 2, Table 3, Table 4, Table 5, Table 6 and Table 7).

Considering E-value estimates and lower bounds, 88.0% and 72.0% of 50 values exceeded nine and were thus in the high zone [67], and 100.0% and 96.0% of lower E-values exceeded the threshold for causality at 1.25 [66] (Table 7).

The order of importance of the primary cannabis metrics was daily cannabis use > herb THC concentration > resin THC concentration (Supplementary Tables S23 and S24).

### 4.2. Choice of Anomalies

The six anomalies analyzed in detail were selected to enable more comprehensive assessment of nervous system disorders overall. Anencephalus has been previously identified by researchers form the Centres for disease control [7] and it was clearly of interest to investigate if this finding would be confirmed in an independent dataset. Severe microcephaly was chosen as it arguably falls on the developmental pathway between anencephalus, reduced head size [17,18] and autistic spectrum disorder, which has previously been noted from prenatal cannabis exposure [14,15,16,69,70]. Further, as this anomaly classification does not exist in the US nomenclature it was of great interest to study it in the European context. Microphthalmia was of interest as it was first identified in the Hawaiian series but not found to be significantly cannabis-associated. It was also identified in a recent US series where it was found to be weakly cannabis associated. The signal in the USA data for both microtia and orofacial clefts was very strong so it was naturally of interest to see how the signal for microphthalmia would perform in the European data. Neural tube anomalies including spina bifida were of great interest as anencephalus is included in this group and had been identified with cannabis exposure, and neural tube anomalies generally had been positively linked with community cannabis exposure in Canada [8].

### 4.3. Qualitative Causal Inference

In 1965, the great epidemiologist A.B. hill described nine criteria which have become standard qualitative criteria against which to judge potentially causal relationships [71]. These criteria were strength of association, consistency amongst studies, specificity, temporality, coherence with known data, biological plausibility, biological dose–response curve, analogy with similar situations elsewhere and experimental confirmation. It is clear that all of these criteria are well fulfilled by the present results. It has been known for several decades that severe central nervous system anomalies were observed in the offspring of animals fed significant doses of cannabinoids during gestation. These defects included anencephalus and hydrocephalus [3,4,5]. Some of the genomic and epigenomic laboratory and clinical evidence is described below.

### 4.4. Quantitative Causal Inference

One of the typical concerns in generalizing the results of observational studies is that the underlying subgroups are not comparable. This issue is well addressed through the technique of choice in causal inferential studies: inverse probability weighting. This technique was deployed in all panel regression studies in this series which effectively overcomes this major interpretational concern and transforms these results from those from an observational study into a pseudorandomized study from which it is entirely proper to draw causal inferences.

The second major concern of such studies is that some extraneous covariate might exist which may account for the effects observed and negate the findings. Use of the E-values sets tight constraints on the associational behavior of such a hypothetical covariate by defining the degree of association required with both the outcome of interest and the exposure of concern of any such extraneous uncontrolled confounder. E-values above nine are generally said to be high [67]. E-values above 1.25 are usually required for causal effects [66]. As noted, 88% of the E-value estimates in the present study were in the high range so that uncontrolled confounding can be effectively discounted in interpreting the present results. Indeed, if the results reported in Supplementary Table S21 are examined closely, it is noted that the median minimum E-value of all six anomalies studied in detail was above 8.7, again giving great assurance that the results reported are indeed robust.

### 4.5. Mechanisms

#### 4.5.1. Morphogen Gradients

Forebrain specification is under the ventralizing control of sonic hedgehog (shh) and fibroblast growth factor 8 (FGF8) released from midline axial structures and opposed by GLI3 dorsally. shh specifies the early forebrain and divides the visual fields into left and right halves [41]. shh greatly stimulates forebrain growth. If the shh signal is blocked, forebrain growth and development is greatly inhibited and the mid-face region does not grow normally. In severe cases, this can lead to holoprosencephaly and sometimes cyclopia (single eye). These data link congenital anomalies affecting the facial region to brain development [41].

Spinal cord formation happens under the tight control of morphogen gradients acting in each of the three dimensional planes [41]. Dorsally high gradients of bone morphogenetic proteins (BMP) and Wnts exist which decline ventrally. Ventrally high gradients of sonic hedgehog exist which decline dorsally. The exterior of the spinal cord releases high gradients of retinoic acid which decline centrally towards the spinal canal. Caudally, there are high gradients of FGF8 and GDF11. Cephalad, there are high gradients of retinoic acid. These morphogen gradients control various gene cassettes (Pax genes dorsally and Nkx genes ventrally) to specific cells exactly in their correct positions including the well known dorsal–ventral split between sensory and motor neurons. Longitudinally, patterns of HOXA and HOXB genes control spinal development.

##### Cannabinoid Inhibition of Morphogens

Cannabis and cannabinoids disrupt many of the above-described morphogen gradients including sonic hedgehog (shh) [29], Wnt signaling [72,73,74,75,76,77], bone morphogenetic proteins [78,79,80], retinoic acid [81,82,83], and fibroblast growth factor [84,85].

Since forebrain development is closely linked with shh signaling [41], shh inhibition by Δ9THC or cannabidiol [29] necessarily implies significant and severe impairment of brain development.

Cannabinoids can also interfere with morphogenetic signaling via epigenomic pathways as described below.

The pattern which emerges from cannabinoid inhibition of these guiding morphogens will depend on the timing and dose exposure of the developing fetus to the xenoteratogen. As noted, the potential implications of disruption of these key controllers of embryological morphogenesis are very serious.

#### 4.5.2. Epigenomic Controls of Brain formation and Neurological Development

The recent paper by Schrott and colleagues in 2021 identified several pathways as importantly disrupted in cannabis dependence in rats and humans including cerebral disorder, neurodevelopmental disorder, agenesis and organismal growth [86]. After 11 weeks of cannabis withdrawal, which is one human sperm cycle in duration, they noted that pathways in hippocampal formation, quantity of pyramidal neurons, organismal death, cognitive impairment, encephalopathy and learning disabilities were increased.

Importantly, cannabis dependence and withdrawal were associated with strong epigenomic links with many key neurotransmitter receptors of both the adult and developing brain including (Table 9; data from [86]) the following: GRIA (Glutamate Ionotropic AMPA Receptor) the predominant excitatory receptor of the brain also involved in long-term depression and potentiation in the hippocampus, mental retardation, depression and neuropathic pain syndromes [87]; GRIK (Glutamate Ionotropic Kainate Receptor) which has role in mental retardation, epilepsy and cold sensation [88]; GRIN (Glutamate Ionotropic NMDA Receptor) which plays critical roles in synaptic plasticity, long-term potentiation and depression and in mental retardation, chronic pain, motor movement and convulsive syndromes and schizophrenia, and Alzheimer’s and Parkinson’s diseases [89]; GRID (Glutamate Ionotropic Delta Type Receptor) is expressed in Purkinje cells of the cerebellum and throughout the brain and when mutated is involved in ataxic movement disorders [90]; GRM (Glutamate Metobotropic Receptor) is implicated in many diseases including schizophrenia, chronic pain syndromes, bipolar disorder, epilepsy and ADHD [91].

Other positive hits include the following: GABR (Gamma-aminobutyric acid receptor) forms the main group of inhibitory receptors in the brain which fine tune and control the action of excitatory receptors. It is also involved in synaptogenesis [92]; GABRA (Gamma-aminobutyric acid receptor Subunit A) is involved in numerous brain functions and importantly in epilepsy [93]; GABRB (Gamma-aminobutyric acid receptor Subunit B) is involved in many brain functions including epilepsy, autism, taste and somatosensory sensation, as a histamine receptor and in synaptogenesis [94]; HTR (5-Hydroxytryptamine Receptors) is involved in many disorders such as depression, schizophrenia, obsessive compulsive disorder, mood, perception, cognition and interaction with many psychoactive substances [95]; DRD (Dopamine Receptor Genes) is related to schizophrenia, addictions, endocrine actions, tremor [96]; DAT1/SLC6A3 (Dopamine Transporter/Solute Carrier Family 6, member 3) is involved in autism, ADHD, depression, bipolar disorder, schizophrenia, epilepsy, addictions, Parkinsonism and movement disorders [97]; OPRM (Opioid Receptor Mu) is the principal target of opioids, endorphins and enkephalins and is also involved in many other addictive drugs actions [98] (it is also involved in heterooligomerization and synaptogenesis); OPRD (Opioid Receptor Delta) is the receptor for enkephalins and is involved in transducing pain perception and immune signaling of certain interleukins (4 and 13) (it is found in the nucleus accumbens, olfactory bulb and neocortex [99]); OXTR (Oxytocin receptor) is involved in parturition and facial recognition, and via projections to the ventral tegmental area, pro-social and pro-sexual stimuli [100]; NRXN (Neurexin Receptor) cell surface ligand of neurexin family ligands includes cerebellins 1 and 2 and forms a key part of synaptic scaffold machinery (it exists in over 3000 spliced isoforms [101] and may be involved in schizophrenia); NLGN (Neuroligin) is a neuronal cell surface protein involved in synaptic formation with neurexins (it is implicated in schizophrenia, autism, mental retardation and pervasive developmental delay and nervous system development [102]); the neurexin–neuroligin ligand–receptor complex is known to play a key role in scaffolding of synapses, synapse formation and synaptic maintenance [103,104,105,106,107]. This system has also been shown to be inhibited by cannabis both directly [103,108] and epigenomically [86].

Details of the epigenomic DNA methylation changes at the opioid receptors are shown in Supplementary Table S25 (data from [86]). Changes are noted to be particularly marked in cannabis dependence.

#### 4.5.3. Downs Syndrome Cell Adhesion Molecule

Supplementary Table S26 (data from [86]) lists 14 epigenomic change annotations for in Downs Syndrome Cell Adhesion Molecule (DSCAM) and in its closely related homologue DSCAM-Like 1 (DSCAML1), proteins which have previously been implicated in central and peripheral nervous system development and involved in neurite guidance and self-avoidance in the brain and retina, and axonal crossing of the midline in the spinal cord [109]. The protein has numerous isoforms and splice variants which allows unique self-coding for cortical and other neurons and thus forms the substrate for self-avoidance guidance cues.

#### 4.5.4. Discs-Large Associated Protein 2

Discs-large associated protein 2 (DLGAP2) is a synaptic protein previously linked with schizophrenia and autism which has been found to be epigenomically altered in cannabis dependence with changes heritable through the paternal line of rats into the nucleus accumbens of offspring [70]. It was also found to be differentially hypomethylated at 17 sites in human sperm [86]. In the present dataset, it was identified at high levels of statistical significance at eight points, as detailed in Supplementary Table S27 (data from [86]).

#### 4.5.5. Retinoic Acid in Forebrain Development—Direct and Epigenomic Effects

A powerful recent study comparing forebrain development in mouse, macaque and human embryos identified strong gradients of retinoic acid (RA) declining from the frontal pole to the premotor cortex as being primarily responsible for the surge in prefrontal cortex (PFC) neuronal numbers, spinogenesis, synaptogenesis, layer 4 lamination with its biomarker RORB and long-range connections to the mediodorsal thalamic nuclei [110]. The RA gradient was maintained by local synthesis by ALDH1A1, transduced by RXR-A, RXR-G and RARB-B receptors and RA was then catabolized towards the premotor cortex by CYP26B1.

As noted above, direct evidence for interaction by cannabinoids with RA has been previously described [81,82,83].

Moreover, there were annotations in the epigenomic study by Schrott and colleagues [86] describing two hits for ALDH1A1 one each in dependence and withdrawal with Bonferroni-adjusted *p*-values of 0.0106 and 0.0291, and nine other hits for members of the ALDH1 family in cannabis dependence and withdrawal. The RA-associated genes cadherin 8 (CDH8) and protocadherin 17 (PCDH17) had one and 156 hits, respectively (Supplementary Table S28, data from [86]). There were five hits for RARB (Bonferroni-adjusted *p*-values from 0.0006 to 0.0248) and one hit each for RXRA, RXRG, RXRA and RORB in cannabis dependence or withdrawal with Bonferroni-adjusted *p*-values from 0.0009 to 0.0191 (Supplementary Table S29, data from [86]).

Whilst CYP26B1 was not identified in this epigenomic screen, other cytochrome P450 isotypes were identified including CYP20A1, CYP27A1, CYP27C1, CYP27C2, and CYP2B7P, CYP2C8, CYP2C18, CYP2C61P and CYP2W1.

Moreover, key amongst the secondary messengers induced by RA in the fetal middle trimester was cerebellin 2 (cbln2), which mediated the massive growth in dendritic spines and synaptogenesis of the human forebrain [111]. RA was found to be a transcription factor acting on the cbln2 enhancer. Cerebellins ligate neurexins 1, 2 and 3 and GRID 1 and 2 which were all highly expressed during midfetal forebrain development. Cannabis has also previously been shown to interfere with neurexin binding [105,107].

#### 4.5.6. Cerebellins

Cerebellin 2 (cbln2) has previously been linked with diverse neurological syndromes including obsessive compulsive disorder, Tourette’s syndrome and schizophrenia and the binding partners of cbln2, neurexins and GRID2 have been linked with autism and schizophrenia [111].

In the epigenomic screen of Schrott, there was one hit for a cbln2 intron in cannabis withdrawal on page 183 with a Bonferroni-adjusted *p*-value of 0.01723 (page 183) [86]. Interestingly, there were 71 hits for cbln4 in from Bonferroni-adjusted *p*-value = 0.0086 (cannabis withdrawal, page 29) and from *p* = 7.73 × 10^−20^ (page 239 in cannabis dependence). Cbln4 has a different distribution in the brain to cbln2 and mainly occupies subcortical and hindbrain sites [112].

#### 4.5.7. Slit-Robo

The robo2-slit1 receptor–ligand system has previously been shown to be key to the exuberant development of the massive human neocortex characterized by very high numbers of neurons and synaptic connections [113,114]. These results were validated by the recent demonstration of the importance of the slit-robo Rho GTPase Activating Protein 2C (SRGAP2C) in a comparative neurodevelopmental study where overexpression of human SRGAP2C in mice led to increased frontal lobe connectivity and improved learning of a task by the modified animals [115]. The slit-robo system can be directly antagonized by cannabinoids [116,117]. Examining both SRGAP2C and its antagonist SRGAP2B in the Schrott data provided the detailed results showing that cannabis also antagonizes this system epigenomically (Supplementary Table S30, data from [86]). Data on annotations relating to slit and robo are reported in Supplementary Table S31 and Table 10, respectively; data from [86].

Other observations from the online supplementary material which accompanied this remarkable paper [86] include the following.

There are 15 references to brain disease made, including glioma (227 genes, page 284), brain lesion (230 genes, page 285), brain tumour (228 genes, page 286), brain size (6 genes, page 351), brain formation (12 genes, page 356), morphology of the nervous system (47 genes, page 300) and forebrain patterning (3 genes, page 333). There are 67 references to neurological disease made, including organismal injury and abnormalities (235 genes, page 281), brain lesion (230 genes, page 285), morphology of enteric ganglia (3 genes, page 308), morphology of spiral ganglia (4 genes, page 312), myelination of sciatic nerve (3 genes, page 314), hair cell morphology (5 genes, page 316) and morphology of stratum pyramidale (3 genes). There are four references to cerebral disorders made, including cerebral disorder (106 genes, page 317), development of the cerebral cortex (6 genes, page 351), head development (47 genes, page 304), cell viability of cerebral cortex cells (4 genes, page 349). Interestingly, amongst the genes controlling cerebral cortex development is GLI3 which is a transducer of the sonic hedgehog pathway [118].

There are 32 references to neurons made, including abnormalities of sensory neurons (7 genes, page 322), neurite growth (26 genes, page 306), abnormal morphology of neurons (26 genes, page 306), proliferation of neural cells (29 genes, page 306), neurogenesis of brain cells (2 genes, page 349), morphology of neurons (35 genes, page 296), neuronal development (43 genes, page 299), neuronal outgrowth (25 genes, page 298), cell movement of brain cells (8 genes, Page 319 and 5 genes, page 347), apoptosis of cortical neurons (4 genes, page 356) and developmental process of synapse formation (15 genes, page 308). There are 11 references made to eyes, including eye formation (25 genes, page 302 and 15 genes, page 328), eye morphology (18 genes, page 308 and 11 genes page 334), morphology of the eye (7 genes, page 318 and 9 genes page 340), eye formation (15 genes, page 328), eye morphogenesis (5 genes, page 338), morphogenesis of camera-type eye (4 genes, page 341) and morphology of eye cells (6 genes, page 342). Genes controlling eye morphology include BMP4 and genes in the sonic hedgehog pathway.

### 4.6. Exponential Genotoxic Effects

A rich literature indicates that both cannabinoid genotoxic effects [20,21,22,23,24,25,26,27,28,29,30] and the metabolic processes upon which they are based [31,32,33,34,35,36] are all subject to exponential dose–response effects which may function clinically as a threshold effect.

Of greater concern is that discrete jumps in the incidence of many congenital anomalies have been described for many anomalies at the highest level of cannabis exposure [1,10,11]. This important finding indicates directly that the concerning findings repeatedly demonstrated in the laboratory are in fact confirmed in the profiles of public health and neonatal seen epidemiologically.

### 4.7. Generalizability

This study is remarkable for its notable consistency with previous reports in animals [3,4,5] and with other epidemiological reports from Australia, Canada and the USA [8,9,10,119,120]. The results also greatly strengthen earlier bivariate reports from Europe and confirm results in the space-time context and formal causal inferential frameworks [119,121]. That is to say that there is an impressive uniformity of many major datasets in various countries around the world which generally indicate similar results. Together, this impressive body of evidence presents a strong and uniform set of results linking cannabinoid exposure with NCA teratological outcomes. A causal relationship between cannabis exposure, on the one hand, and NCA teratology, on the other, is further supported by the many mechanistic pathways which form a key and pivotal plank of formal causal algorithms of establishing causality [71] and further indicating the causal nature of this link.

Of particular importance in this discussion is the evidently graded series of NCAs from mildly impaired intellectual development to microcephaly, to severe microcephaly to anencephaly which demonstrates a clear and graded spectrum of adverse effects of cannabinoid exposure on infant and child neurological development.

In view of this impressive and remarkably uniform body of work, it seems clear that the relationship described is causal in nature. Because of this notable confluence of large epidemiological datasets on this issue, the strength of both the statistical and mechanistic arguments for causality and the concordance with data from preclinical models, we feel that this relationship is widely generalizable to other places wherever data of sufficient size and quality exist for it to be reliably assessed.

### 4.8. Strengths and Limitations

This study had a number of strengths including the use of one of the world’s largest and most comprehensive congenital anomaly datasets; the use of advanced statistical modelling; the use of inverse probability weighting and E-values to engage the techniques for formal quantitative causal inference and transfer the analysis from an observational study to a pseudorandomized study; the liberal use of multi-paneled maps and graphs to display multiple covariates across time and space; the use of bivariate maps, which is unusual, and the provision of extensive supplementary material, online resources, data and computational code. Ranger regression was used for formal variable selection. Like many epidemiological studies, the present work did not have access to individual cannabis exposure data. Additionally, some of the data, particularly those relating to daily cannabis use, had to be interpolated due to severe missing data problems. This limitation should be borne in mind when interpreting results for relevant parameters.

### 4.9. Future Directions

The present strong and now widely replicated results reported herein and in similar comparable studies internationally clearly place a major spotlight on the whole issue of the impacts of cannabinoids on neural development. Hence, the impact of the present report is to increase the research importance of these issues and justify further detailed studies in these areas. Further fine-resolution spatiotemporal and causal inferential epidemiological studies need to be performed. Mechanistic and particularly epigenomic studies need to be explored in detail to further understand these effects and are all strongly indicated.

## 5. Conclusions

This study found that nine of the eleven NCAs in this European dataset were closely related to metrics of cannabis exposure on bivariate analysis and this relationship persisted after adjustment in all six of the NCAs selected for further detailed multivariable analysis. In this regard, these results are consistent with those from Canada, Hawaii, Colorado and the USA [1,10,11] and also with previous reports from CDC [7]. The major tools of formal quantitative causal inference were widely employed in this study. Inverse probability weighting of all panel models transferred the findings from those of a merely observational study into a pseudorandomized study from which it is quite appropriate to draw casual inferences. Collected E-values were predominantly in the high range; effectively excluding uncontrolled confounding was a possible explanation for these results. Therefore, these results fulfilled quantitative epidemiological criteria of causality. Beyond the very concerning findings of a number of NCAs closely related to metrics of cannabis exposure, findings raise two particular concerns. The first relates to interference with brain development during in utero life, which is typically a long-term disability from which recovery is difficult and the degree of disability severe. The second relates to the now well documented exponential dose–response relationships of cannabinoids both in the laboratory [22,23,24,25,26,30,35] and in a number of recent epidemiological studies [1,10,11]. Both these concerns strongly indicate that penetration of cannabinoids into the community should rationally be tightly restricted if we are to seriously steward our responsibilities as custodians of the human brain, genome and epigenome for the generations to come.

## Figures and Tables

**Table 1:** Multivariate Geospatial Analysis for Central Nervous System Disorders.

Parameter Values			Model Parameters		
Parameter	Estimate (C.I.)	*p*-Value	Parameter	Value	Significance
*Additive*					
*Rate ~ Tobacco + Alcohol + LM.Cannabis_x_Herb.THC_x_Daily.Interpol. + LM.Cannabis_x_Resin.THC_x_Daily.Interpol. + Daily.Interpol. + LM.Cannabis_x_Resin.THC + Amphetamines + Cocaine + Income)*					
Tobacco	0.05 (0.02, 0.09)	0.0010	psi	0.8032	<2.2 × 10^−16^
LM.Cannabis_x_Herb.THC	5.23 (1.82, 8.64)	0.0027	rho	0.62462	4.19 × 10^−10^
Income	0 (0, 0)	0.0005	lambda	−0.7094	8.38 × 10^−15^
*Interactive*					
Rate ~ Tobacco * LM.Cannabis_x_Herb.THC_x_Daily.Interpol. * LM.Cannabis_x_Resin.THC_x_Daily.Interpol. + Daily.Interpol. + LM.Cannabis_x_Herb.THC + Alcohol + Amphetamines + Cocaine + Income					
LM.Cannabis_x_Herb.THC_x_Daily.Interpol.	−15.7 (−27.56, −3.84)	0.0094	psi	0.79839	<2.2 × 10^−16^
LM.Cannabis_x_Resin.THC_x_Daily.Interpol.	5.18 (0.18, 10.18)	0.04239	rho	0.70837	<2.2 × 10^−16^
Alcohol	0.12 (0.07, 0.18)	4.14 × 10^−5^	lambda	−0.72814	<2.2 × 10^−16^
Cocaine	0.15 (0.02, 0.28)	0.0261			
Income	0 (0, 0)	0.0011			
Tobacco: LM.Cannabis_x_Herb.THC_x_Daily.Interpol.	0.68 (0.21, 1.16)	0.0050			
Tobacco: LM.Cannabis_x_Resin.THC_x_Daily.Interpol.	−0.22 (−0.42, −0.02)	0.0314			
*1 Lag*					
Rate ~ Tobacco * LM.Cannabis_x_Herb.THC_x_Daily.Interpol. + LM.Cannabis_x_Resin.THC_x_Daily.Interpol. * LM.Cannabis_x_Herb.THC + Alcohol + Daily.Interpol. + Amphetamines + Cocaine + Income					
Tobacco	0.06 (0.03, 0.1)	0.0008	psi	0.8217	<2.2 × 10^−16^
Income	0 (0, 0)	0.0029	rho	0.6356	2.40 × 10^−9^
			lambda	−0.71467	1.39 × 10^−13^
*2 Lags*					
Rate ~ Tobacco * LM.Cannabis_x_Herb.THC_x_Daily.Interpol. + LM.Cannabis_x_Resin.THC_x_Daily.Interpol. * LM.Cannabis_x_Herb.THC + Alcohol + Daily.Interpol. + Amphetamines + Cocaine + Income					
Tobacco	0.08 (0.04, 0.12)	3.23 × 10^−5^	psi	0.75493	<2.2 × 10^−16^
Income	0 (0, 0)	0.0004	rho	0.41765	0.0521
			lambda	−0.4742	0.0225

Abbreviations: LM.Cannabis—last month cannabis use; Herb.THC—THC concentration of cannabis herb; Resin.THC—THC concentration of cannabis resin; Daily.Interpol.—Daily cannabis use interpolated.

**Table 2:** Multivariate Geospatial Analysis for Anencephalus.

Parameter Values			Model Parameters		
Parameter	Estimate (C.I.)	*p*-Value	Parameter	Value	Significance
*Additive*					
*Rate ~ Tobacco + Alcohol + LM.Cannabis_x_Herb.THC_x_Daily.Interpol. + LM.Cannabis_x_Resin.THC_x_Daily.Interpol. + Daily.Interpol. + LM.Cannabis_x_Resin.THC + Amphetamines + Cocaine + Income)*					
Tobacco	0.06 (0.02, 0.11)	0.0050	psi	0.68084	<2.2 × 10^−16^
Income	0 (0, 0)	0.0001	rho	0.65932	8.56 × 10^−12^
			lambda	−0.77182	<2.2 × 10^−16^
*Interactive*					
Rate ~ Tobacco * LM.Cannabis_x_Resin.THC_x_Daily.Interpol. + Daily.Interpol. + LM.Cannabis_x_Resin.THC + Resin + Alcohol + Amphetamines + Cocaine + Income					
Tobacco	0.09 (0.04, 0.13)	0.0002	psi	0.5578	7.07 × 10^−6^
LM.Cannabis_x_Resin.THC	2.7 (0.66, 4.74)	0.0092	rho	−0.6788	9.48 × 10^−8^
Income	0 (0, 0)	0.0002	lambda	0.5255	0.000365
Tobacco: LM.Cannabis_x_Resin.THC_x_Daily.Interpol.	−0.07 (−0.15, 0)	0.0466			
*1 Lag*					
Rate ~ Tobacco * LM.Cannabis_x_Resin.THC_x_Daily.Interpol. + LM.Cannabis_x_Resin.THC + Resin + Daily.Interpol. + Alcohol + Amphetamines + Cocaine + Income					
Tobacco	0.07 (0.03, 0.12)	0.0004	psi	0.6289	2.34 × 10^−14^
LM.Cannabis_x_Resin.THC	5.5 (3.44, 7.56)	1.53 × 10^−7^	rho	0.6326	1.57 × 10^−8^
Income	0 (0, 0)	0.0005	lambda	−0.635	1.69 × 10^−8^
Tobacco: LM.Cannabis_x_Resin.THC_x_Daily.Interpol.	−0.08 (−0.13, −0.02)	0.0036			
*2 Lags*					
Rate ~ Tobacco * LM.Cannabis_x_Resin.THC_x_Daily.Interpol. + LM.Cannabis_x_Resin.THC * Resin + Daily.Interpol. + Alcohol + Amphetamines + Cocaine + Income					
Resin	3.48 (2.17, 4.79)	1.97 × 10^−7^	psi	0.88545	<2.2 × 10^−16^
			rho	0.59616	2.10 × 10^−6^
			lambda	−0.6442	1.76 × 10^−7^

Abbreviations—Please see Table 1.

**Table 3:** Multivariate Geospatial Analysis for Severe Microcephalus.

Parameter Values			Model Parameters		
Parameter	Estimate (C.I.)	*p*-Value	Parameter	Value	Significance
*Additive*					
*Rate ~ Tobacco + Alcohol + LM.Cannabis_x_Herb.THC_x_Daily.Interpol. + LM.Cannabis_x_Resin.THC_x_Daily.Interpol. + Daily.Interpol. + LM.Cannabis_x_Resin.THC + Amphetamines + Cocaine + Income)*					
Alcohol	0.22 (0.14, 0.3)	2.14 × 10^−8^	psi	0.32056	0.00128
Daily.Interpol.	37.8 (21.83, 53.77)	3.65 × 10^−6^	rho	0.53742	1.16 × 10^−5^
Amphetamines	−0.25 (−0.43, −0.06)	0.01052	lambda	−0.4586	0.000461
Income	0 (0, 0)	0.00335			
*Interactive*					
Rate ~ Tobacco * LM.Cannabis_x_Herb.THC_x_Daily.Interpol. + Daily.Interpol. + LM.Cannabis_x_Resin.THC + Herb + Alcohol + Herb + Amphetamines + Cocaine + Income					
Resin	37.8 (21.83, 53.77)	3.65 × 10^−6^	psi	0.32056	0.00128
Alcohol	0.22 (0.14, 0.3)	2.14 × 10^−8^	rho	0.53742	1.17 × 10^−5^
Amphetamines	−0.25 (−0.43, −0.06)	0.01052	lambda	−0.4586	0.000463
Income	0 (0, 0)	0.00335			
*2 Lags*					
Rate ~ Tobacco * LM.Cannabis_x_Herb.THC_x_Daily.Interpol. + Daily.Interpol. + LM.Cannabis_x_Herb.THC + Alcohol + Amphetamines + Cocaine + Income					
Tobacco	0.15 (0.09, 0.21)	1.98 × 10^−6^	psi	0.238	0.0374
LM.Cannabis_x_Herb.THC_x_Daily.Interpol.	45.4 (17.96, 72.84)	0.00117	rho	−0.2833	0.2029
Alcohol	0.14 (0.06, 0.23)	0.00132	lambda	0.2726	0.145
Amphetamines	−0.36 (−0.58, −0.14)	0.00149			
Income	0 (0, 0)	3.26 × 10^−7^			
Tobacco: LM.Cannabis_x_Herb.THC_x_Daily.Interpol.	−1.68 (−2.76, −0.6)	0.00234			

Abbreviations—Please see Table 1.

**Table 4:** Multivariate Geospatial Analysis for Microphthalmia.

Parameter Values			Model Parameters		
Parameter	Estimate (C.I.)	*p*-Value	Parameter	Value	Significance
*Additive*					
*Rate ~ Tobacco + Alcohol + LM.Cannabis_x_Herb.THC_x_Daily.Interpol. + LM.Cannabis_x_Resin.THC_x_Daily.Interpol. + Daily.Interpol. + LM.Cannabis_x_Resin.THC + Amphetamines + Cocaine + Income)*					
Tobacco	0.03 (0.01, 0.06)	0.01993	psi	0.1805	0.085341
LM.Cannabis_x_Herb.THC_x_Daily.Interpol.	1.32 (0.11, 2.53)	0.03192	rho	0.5289	0.000136
Income	0 (0, 0)	0.00654	lambda	−0.51	0.000565
*Interactive*					
Rate ~ Tobacco * Resin + Daily.Interpol. + Herb + LM.Cannabis_x_Herb.THC_x_Daily.Interpol. + Alcohol + Amphetamines + Cocaine + Income					
Resin	−6.17 (−9.17, −3.17)	5.26 × 10^−5^	rho	−0.5311	0.000340
Cocaine	0.23 (0.09, 0.36)	0.00111	lambda	0.4738	0.000591
Income	0 (0, 0)	0.00197			
Tobacco: Resin	0.24 (0.13, 0.35)	3.76 × 10^−5^			
*1 Lag*					
Rate ~ Tobacco * Resin + Daily.Interpol. + Herb + LM.Cannabis_x_Herb.THC_x_Daily.Interpol. + Alcohol + Amphetamines + Cocaine + Income					
Resin	−11.7 (−16.25, −7.15)	4.47 × 10^−7^	psi	−0.03769	0.748
LM.Cannabis_x_Herb.THC_x_Daily.Interpol.	2.71 (1.02, 4.4)	0.001741	rho	0.65087	2.06 × 10^−11^
Alcohol	−0.12 (−0.18, −0.05)	0.000918	lambda	−0.64586	2.57 × 10^−11^
Cocaine	−0.2 (−0.4, 0)	0.047988			
Income	0 (0, 0)	1.55 × 10^−6^			
Tobacco: Resin	0.41 (0.25, 0.57)	5.18 × 10^−7^			
*2 Lags*					
Rate ~ Tobacco * Resin + Daily.Interpol. + Herb + LM.Cannabis_x_Herb.THC_x_Daily.Interpol. + Alcohol + Amphetamines + Cocaine + Income					
Resin	−11.7 (−15.82, −7.58)	2.73 × 10^−8^	rho	0.5539	3.46 × 10^−5^
Alcohol	−0.1 (−0.17, −0.03)	0.00318	lambda	−0.6037	1.21 × 10^−6^
Income	0 (0, 0)	9.17 × 10^−7^			
Tobacco: Resin	0.45 (0.29, 0.61)	2.13 × 10^−8^			

Abbreviations—Please see Table 1.

**Table 5:** Multivariate Geospatial Analysis for Neural Tube Defects.

Parameter Values			Model Parameters		
Parameter	Estimate (C.I.)	*p*-Value	Parameter	Value	Significance
*Additive*					
*Rate ~ Tobacco + Alcohol + LM.Cannabis_x_Herb.THC_x_Daily.Interpol. + LM.Cannabis_x_Resin.THC_x_Daily.Interpol. + Daily.Interpol. + LM.Cannabis_x_Resin.THC + Amphetamines + Cocaine + Income)*					
Tobacco	0.07 (0.04, 0.11)	1.26 × 10^−4^	psi	0.6859	<2 × 10^−16^
Herb	4.26 (1.26, 7.26)	0.0054	rho	0.5583	2.33 × 10^−5^
Income	0 (0, 0)	0.0007	lambda	−0.6312	1.70 × 10^−7^
*Interactive*					
Rate ~ Tobacco + Daily.Interpol. * LM.Cannabis_x_Resin.THC_x_Daily.Interpol. + LM.Cannabis_x_Resin.THC + Resin + Alcohol + Amphetamines + Cocaine + Income					
Tobacco	0.08 (0.04, 0.12)	2.27 × 10^−5^	psi	0.6508	<2 × 10^−16^
Income	0 (0, 0)	5.01 × 10^−5^	rho	0.5338	0.0003
			lambda	−0.6369	2.75 × 10^−6^
*1 Lag*					
Rate ~ Tobacco + Daily.Interpol. * LM.Cannabis_x_Resin.THC_x_Daily.Interpol. + LM.Cannabis_x_Resin.THC + Alcohol + Daily.Interpol. + Amphetamines + Cocaine + Income					
Tobacco	0.07 (0.03, 0.11)	0.0002	psi	0.5946	8.78 × 10^−12^
LM.Cannabis_x_Resin.THC_x_Daily.Interpol.	−1.15 (−2.29, −0.01)	0.0485	rho	0.4815	0.0063
LM.Cannabis_x_Resin.THC	3.59 (1.73, 5.45)	0.0002	lambda	−0.5681	0.0003
Income	0 (0, 0)	0.0007			
*2 Lags*					
Rate ~ Tobacco + Daily.Interpol. * LM.Cannabis_x_Resin.THC_x_Daily.Interpol. + LM.Cannabis_x_Resin.THC + Alcohol + Daily.Interpol. + Amphetamines + Cocaine + Income					
Tobacco	0.1 (0.05, 0.15)	3.49 × 10^−5^	psi	0.6627	1.03 × 10^−14^
LM.Cannabis_x_Resin.THC_x_Daily.Interpol.	2.28 (0.35, 4.21)	0.0208	rho	0.5557	9.20 × 10^−5^
LM.Cannabis_x_Resin.THC	−2.54 (−4.74, −0.34)	0.0239	lambda	−0.6187	6.85 × 10^−6^
Income	0 (0, 0)	4.71 × 10^−5^			

Abbreviations—Please see Table 1.

**Table 6:** Multivariate Geospatial Analysis for Spina Bifida.

Parameter Values			Model Parameters		
Parameter	Estimate (C.I.)	*p*-Value	Parameter	Value	Significance
*Additive*					
*Rate ~ Tobacco + Alcohol + LM.Cannabis_x_Herb.THC_x_Daily.Interpol. + LM.Cannabis_x_Resin.THC_x_Daily.Interpol. + Daily.Interpol. + LM.Cannabis_x_Resin.THC + Amphetamines + Cocaine + Income)*					
Tobacco	0.08 (0.05, 0.11)	2.21 × 10^−8^	psi	0.3329	0.0006
Herb	3.28 (1.18, 5.38)	0.0021	rho	−0.7510	<2.2 × 10^−16^
Amphetamines	0.13 (0.02, 0.24)	0.0201	lambda	0.5524	5.98 × 10^−8^
Income	0 (0, 0)	2.44 × 10^−5^			
*Interactive*					
Rate ~ Tobacco + Herb * Resin + LM.Cannabis_x_Resin.THC_x_Daily.Interpol. + LM.Cannabis_x_Resin.THC + Alcohol + Amphetamines + Cocaine + Income					
Tobacco	0.09 (0.07, 0.11)	1.06 × 10^−15^	rho	−0.7547	
LM.Cannabis_x_Resin.THC_x_Daily.Interpol.	−0.42 (−0.75, −0.09)	0.0131	lambda	0.4883	<2.2 × 10^−16^
Income	0 (0, 0)	5.11 × 10^−11^			4.70 × 10^−6^
Herb: Resin	17.1 (11.12, 23.08)	1.92 × 10^−8^			
*1 Lag*					
Rate ~ Tobacco + Herb * Resin + LM.Cannabis_x_Resin.THC + LM.Cannabis_x_Resin.THC_x_Daily.Interpol. + Alcohol + Daily.Interpol. + Amphetamines + Cocaine + Income					
Tobacco	0.09 (0.06, 0.11)	5.79 × 10^−15^	rho	−0.7561	<2.2 × 10^−16^
LM.Cannabis_x_Resin.THC	4.01 (2.86, 5.16)	9.76 × 10^−12^	lambda	0.4818	2.44 × 10^−6^
LM.Cannabis_x_Resin.THC_x_Daily.Interpol.	−2.15 (−2.95, −1.35)	1.39 × 10^−7^			
Cocaine	−0.16 (−0.29, −0.02)	0.0238			
Income	0 (0, 0)	7.63 × 10^−15^			
*2 Lags*					
Rate ~ Tobacco + Herb * Resin + LM.Cannabis_x_Resin.THC + LM.Cannabis_x_Resin.THC_x_Daily.Interpol. + Alcohol + Daily.Interpol. + Amphetamines + Cocaine + Income					
Tobacco	0.06 (0.03, 0.09)	0.0002	psi	0.2973	0.00632
Resin	−3.51 (−6.18, −0.84)	0.0096	rho	0.6272	3.52 × 10^−8^
LM.Cannabis_x_Resin.THC_x_Daily.Interpol.	1.58 (0.37, 2.79)	0.0102	lambda	−0.6951	8.93 × 10^−10^
Daily.Interpol.	−23.4 (−41.55, −5.25)	0.0115			
Income	0 (0, 0)	0.0004			
Herb: Resin	26.5 (3.96, 49.04)	0.0209			

Abbreviations—Please see Table 1.

**Table 7:** List of All E-Values.

No.	E-Value Estimate	Lower Bound E-Value
1	1.89 × 10^35^	1.04 × 10^29^
2	1.89 × 10^35^	1.04 × 10^29^
3	1.89 × 10^35^	1.04 × 10^29^
4	1.89 × 10^35^	1.04 × 10^29^
5	6.54 × 10^34^	3.32 × 10^25^
6	6.54 × 10^34^	3.32 × 10^25^
7	5.85 × 10^29^	9.39 × 10^20^
8	5.85 × 10^29^	9.39 × 10^20^
1	1.56 × 10^22^	2.92 × 10^9^
9	1.56 × 10^22^	2.92 × 10^9^
10	2.71 × 10^17^	1.45 × 10^6^
11	2.71 × 10^17^	1.45 × 10^6^
12	9.28 × 10^11^	1.11 × 10^6^
13	9.28 × 10^11^	1.11 × 10^6^
14	1.82 × 10^7^	7.92 × 10^4^
15	1.82 × 10^7^	7.92 × 10^4^
16	5.31 × 10^5^	2.43 × 10^4^
17	5.31 × 10^5^	2.43 × 10^4^
18	4.84 × 10^5^	868.15
19	4.84 × 10^5^	868.15
20	1.35 × 10^4^	455.47
21	1.35 × 10^4^	455.47
22	4.98 × 10^3^	81.79
23	4.98 × 10^3^	81.79
24	727.65	44.80
25	727.65	44.80
26	727.65	38.03
27	727.65	38.03
28	272.41	28.95
29	272.41	28.95
30	184.48	28.95
31	184.48	28.95
32	49.08	22.02
33	49.08	22.02
34	49.08	22.02
35	49.08	22.02
36	35.92	8.79
37	35.92	8.79
38	35.92	8.79
39	35.92	8.79
40	23.23	1.64
41	23.23	1.64
42	23.23	1.60
43	23.23	1.60
44	2.17	1.60
45	2.17	1.60
46	2.11	1.44
47	2.11	1.44
48	1.81	1.19
49	1.81	1.19

**Table 8:** Summary of E-Values by Anomaly.

Anomaly	Number	Mean Minimum E-Value	Median Minimum E-Value	Min Minimum E-Value	Max Minimum E-Value	Mean E-Value Estimate	Median E-Value Estimate	Min E-Value Estimate	Max E-Value Estimate
Severe Microcephalus	6	6.93 × 10^28^	1.04 × 10^29^	1.11 × 10^6^	1.04 × 10^29^	1.26 × 10^35^	1.89 × 10^35^	1.82 × 10^7^	1.89 × 10^35^
Microphthalmos	8	8.30 × 10^24^	4.70 × 10^20^	1.44	3.32 × 10^25^	1.64 × 10^34^	2.93 × 10^29^	2.11	6.54 × 10^34^
Nervous	10	5.84 × 10^8^	7.92 × 10^4^	38.03	2.92 × 10^9^	3.12 × 10^21^	9.28 × 10^11^	272.41	1.56 × 10^22^
Neural Tube Defects	8	231.81	28.95	1.19	868.15	1269.9925	49.08	1.81	4980.00
Anencephalus	6	41.94	22.02	22.02	81.79	85.44	35.92	35.92	184.48
Spina Bifida	12	86.84	8.79	1.6	455.47	9.10 × 10^4^	727.65	23.23	5.31 × 10^5^

**Table 9:** Epigenomic Data for Key Synaptic EWAS Hits, Data from Schrott [86].

Nearest Gene Name	Number of Annotations	Page	Chromosome	Status	Nearest Gene	Distance to Nearest Gene	Relative Location	*p*-Value	P-Adjust
Glutamate/GABA Receptors									
GRIA	132	8	11	Dependence	ENSG00000152578	0	Intron	1.96 × 10^−8^	0.000888
GRIK	165	24	1	Dependence	ENSG00000163873	0	Intron	5.18 × 10^−7^	0.005036
GRIN	26	14	16	Dependence	ENSG00000183454	0	Intron	1.18 × 10^−7^	0.002371
GRID	11	7	4	Dependence	ENSG00000152208	0	Intron	1.10 × 10^−8^	0.000694
GRM	122	10	3	Dependence	ENSG00000196277	0	Intron	4.74 × 10^−8^	0.001439
GABR	143	18	4	Dependence	ENSG00000151834	0	Intron	2.72 × 10^−7^	0.003664
GABRA	142	52	4	Dependence	ENSG00000151834	0	Intron	2.74 × 10^−6^	0.011395
GABRB	22	18	4	Dependence	ENSG00000163288	0	Intron	2.72 × 10^−7^	0.003664
Other Receptors									
HTR	85	36	X	Dependence	ENSG00000147246	0	Intron	1.31 × 10^−6^	0.007988
DRD	17	33	5	Dependence	ENSG00000184845	0	Intron	1.06 × 10^−6^	0.007226
DAT	52	214	4	Withdrawal	ENSG00000109576	0	Intron	1.18 × 10^−5^	0.024539
MOR	379	68	X	Dependence	ENSG00000231154	0	Intron	4.87 × 10^−6^	0.015091
DOR/KOR	0	-	-	-	-	-	-	-	-
OXTR	7	55	3	Dependence	ENSG00000180914	0	Intron	3.10 × 10^−6^	0.012043
Synaptic Scaffolding									
NXN	10	32	1	Dependence	ENSG00000226693	0	Intron	1.03 × 10^−6^	0.007159
NLG	10	48	3	Dependence	ENSG00000169760	0	Intron	2.35 × 10^−6^	0.010632

**Table 10:** Epigenomic Data for Robo EWAS Hits, Data from Schrott [86].

**Nearest Gene Name**	**Page**	**Chromosome**	**Status**	**Nearest Gene**	**Distance to Nearest Gene**	**Relative Location**	***p*-Value**	***p*-Adjust**
ROBO1								
ROBO1	22	3	Dependence	ENCG00000169855	0	Intron	4.08 × 10^−7^	0.004487
ROBO1	34	3	Dependence	ENCG00000169855	0	Intron	1.10 × 10^−6^	0.007347
ROBO1	179	3	Withdrawal	ENCG00000169855	0	Intron	4.75 × 10^−6^	0.016077
ROBO1	75	3	Dependence	ENCG00000169855	0	Intron	5.83 × 10^−6^	0.016430
ROBO1	82	3	Dependence	ENCG00000169855	1176	Downstream	7.09 × 10^−6^	0.017986
ROBO1	103	3	Dependence	ENCG00000169855	0	Intron	1.10 × 10^−5^	0.022061
ROBO1	118	3	Dependence	ENCG00000169855	90264	Upstream	1.46 × 10^−5^	0.025364
ROBO2								
ROBO2	8	3	Dependence	ENCG00000185008	0	Intron	1.72 × 10^−8^	0.000855
ROBO2	17	3	Dependence	ENCG00000185008	0	Intron	2.30 × 10^−7^	0.003353
ROBO2	139	3	Withdrawal	ENCG00000185008	0	Intron	4.68 × 10^−7^	0.005298
ROBO2	149	3	Withdrawal	ENCG00000185008	0	Intron	1.20 × 10^−6^	0.008443
ROBO2	167	3	Withdrawal	ENCG00000185008	0	Intron	3.15 × 10^−6^	0.013257
ROBO2	66	3	Dependence	ENCG00000185008	0	Intron	4.52 × 10^−6^	0.014551
ROBO2	200	3	Withdrawal	ENCG00000185008	0	Intron	8.80 × 10^−6^	0.021641
ROBO2	204	3	Withdrawal	ENCG00000185008	0	Intron	9.48 × 10^−6^	0.022315
ROBO2	106	3	Dependence	ENCG00000185008	0	Intron	1.16 × 10^−5^	0.022679
ROBO2	223	3	Withdrawal	ENCG00000185008	0	Intron	1.40 × 10^−5^	0.026641
ROBO2	149	3	Withdrawal	ENCG00000185008	0	Intron	1.20 × 10^−6^	0.084427
ROBO4								
ROBO4	230	11	Withdrawal	ENCG00000154133	0	Intron	1.59 × 10^−5^	0.028295
**Nearest Gene Name**	**Page**	**Chromosome**	**Status**	**Nearest Gene**	**Fold Enrichment**	***p*-Value**	**P-Bonferroni**	**P-FDR**
ROBO1	237	3	Dependence	ENCG00000169855	1.7082	1.12 × 10^−2^	0.9504	0.001219
ROBO2	237	3	Dependence	ENCG00000185008	1.7082	1.12 × 10^−2^	0.9504	0.001219

## Data Availability

All data generated or analyzed during this study are included in this published article and its supplementary information files. Data along with the relevant R code have been made publicly available on the Mendeley Database Repository and can be accessed from these URLs: 10.17632/vd6mt5r5jm.1 and 10.17632/gr5ntsbp7p.1.

## Supplementary Materials

The following supporting information can be downloaded at: https://www.mdpi.com/article/10.3390/ijerph20010441/s1, Supplementary Figures and Tables have been made available on line together with this paper on the journal website.

## References

[1] Reece A.S., Hulse G.K. (2022). Cannabinoid- and Substance- Relationships of European Congenital Anomaly Patterns: A Space-Time Panel Regression and Causal Inferential Study. Environ. Epigenet..

[2] Reece A.S., Hulse G.K. (2022). Congenital Anomaly Epidemiological Correlates of Δ8THC across USA 2003–2016: Panel Regression and Causal Inferential Study. Environ. Epigenet..

[3] Graham J.D.P. (1976). Cannabis and Health. Cannabis and Health.

[4] Geber W.F., Schramm L.C. (1969). Teratogenicity of marihuana extract as influenced by plant origin and seasonal variation. Arch. Int. Pharmacodyn. Ther..

[5] Geber W.F., Schramm L.C. (1969). Effect of marihuana extract on fetal hamsters and rabbits. Toxicol. Appl. Pharmacol..

[6] Forrester M.B., Merz R.D. (2007). Risk of selected birth defects with prenatal illicit drug use, Hawaii, 1986–2002. J. Toxicol. Environ. Health.

[7] Van Gelder M.M.H.J., Donders A.R.T., Devine O., Roeleveld N., Reefhuis J. (2014). Using bayesian models to assess the effects of under-reporting of cannabis use on the association with birth defects, national birth defects prevention study, 1997–2005. Paediatr. Perinat. Epidemiol..

[8] Reece A.S., Hulse G.K. (2019). Cannabis Consumption Patterns Explain the East-West Gradient in Canadian Neural Tube Defect Incidence: An Ecological Study. Glob. Pediatr. Health.

[9] Reece A.S., Hulse G.K. (2020). Broad Spectrum epidemiological contribution of cannabis and other substances to the teratological profile of northern New South Wales: Geospatial and causal inference analysis. BMC Pharmacol. Toxicol..

[10] Reece A.S., Hulse G.K. (2022). Geotemporospatial and causal inference epidemiological analysis of US survey and overview of cannabis, cannabidiol and cannabinoid genotoxicity in relation to congenital anomalies 2001–2015. BMC Pediatr..

[11] Reece A.S., Hulse G.K. (2020). Cannabis in Pregnancy–Rejoinder, Exposition and Cautionary Tales. Psychiatr. Times.

[12] Reece A.S., Hulse G.K. (2021). Quadruple convergence–rising cannabis prevalence, intensity, concentration and use disorder treatment. Lancet Reg. Health-Eur..

[13] Manthey J., Freeman T.P., Kilian C., Lopez-Pelayo H., Rehm J. (2021). Public health monitoring of cannabis use in Europe: Prevalence of use, cannabis potency, and treatment rates. Lancet Reg. Health-Eur..

[14] Reece A.S., Hulse G.K. (2019). Epidemiological Associations of Various Substances and Multiple Cannabinoids with Autism in USA. Clin. Pediatr. Open Access.

[15] Reece A.S., Hulse G.K. (2019). Effect of Cannabis Legalization on US Autism Incidence and Medium Term Projections. Clin. Pediatr. Open Access.

[16] Reece A.S., Hulse G.K. (2019). Gastroschisis and Autism-Dual Canaries in the Californian Coalmine. JAMA Surg..

[17] Volkow N.D., Han B., Compton W.M., Blanco C. (2017). Marijuana Use During Stages of Pregnancy in the United States. Ann. Intern. Med..

[18] Volkow N.D., Compton W.M., Wargo E.M. (2017). The Risks of Marijuana Use During Pregnancy. JAMA.

[19] Volkow N.D., Han B., Compton W.M., McCance-Katz E.F. (2019). Self-reported Medical and Nonmedical Cannabis Use among Pregnant Women in the United States. JAMA.

[20] Hölzel B.N., Pfannkuche K., Allner B., Allner H.T., Hescheler J., Derichsweiler D., Hollert H., Schiwy A., Brendt J., Schaffeld M. (2020). Following the adverse outcome pathway from micronucleus to cancer using H2B-eGFP transgenic healthy stem cells. Arch. Toxicol..

[21] Russo C., Ferk F., Misik M., Ropek N., Nersesyan A., Mejri D., Holzmann K., Lavorgna M., Isidori M., Knasmuller S. (2018). Low doses of widely consumed cannabinoids (cannabidiol and cannabidivarin) cause DNA damage and chromosomal aberrations in human-derived cells. Arch. Toxicol..

[22] Tahir S.K., Trogadis J.E., Stevens J.K., Zimmerman A.M. (1992). Cytoskeletal organization following cannabinoid treatment in undifferentiated and differentiated PC12 cells. Biochem. Cell Biol..

[23] Vela G., Martin S., Garcia-Gil L., Crespo J.A., Ruiz-Gayo M., Fernandez-Ruiz J.J., Garcia-Lecumberri C., Pelaprat D., Fuentes J.A., Ramos J.A. (1998). Maternal exposure to delta9-tetrahydrocannabinol facilitates morphine self-administration behavior and changes regional binding to central mu opioid receptors in adult offspring female rats. Brain Res..

[24] Busch F.W., Seid D.A., Wei E.T. (1979). Mutagenic activity of marihuana smoke condensates. Cancer Lett..

[25] Koller V.J., Ferk F., Al-Serori H., Misik M., Nersesyan A., Auwarter V., Grummt T., Knasmuller S. (2015). Genotoxic properties of representatives of alkylindazoles and aminoalkyl-indoles which are consumed as synthetic cannabinoids. Food Chem. Toxicol..

[26] Zimmerman A.M., Raj A.Y. (1980). Influence of cannabinoids on somatic cells in vivo. Pharmacology.

[27] Shoyama Y., Sugawa C., Tanaka H., Morimoto S. (2008). Cannabinoids act as necrosis-inducing factors in Cannabis sativa. Plant Signal Behav..

[28] Price P.J., Suk W.A., Spahn G.J., Freeman A.E. (1972). Transformation of Fischer rat embryo cells by the combined action of murine leukemia virus and (-)-trans-9-tetrahydrocannabinol. Proc. Soc. Exp. Biol. Med..

[29] Fish E.W., Murdaugh L.B., Zhang C., Boschen K.E., Boa-Amponsem O., Mendoza-Romero H.N., Tarpley M., Chdid L., Mukhopadhyay S., Cole G.J. (2019). Cannabinoids Exacerbate Alcohol Teratogenesis by a CB1-Hedgehog Interaction. Sci. Rep..

[30] Koller V.J., Auwarter V., Grummt T., Moosmann B., Misik M., Knasmuller S. (2014). Investigation of the in vitro toxicological properties of the synthetic cannabimimetic drug CP-47,497-C8. Toxicol. Appl. Pharmacol..

[31] Sarafian T.A., Habib N., Oldham M., Seeram N., Lee R.P., Lin L., Tashkin D.P., Roth M.D. (2006). Inhaled marijuana smoke disrupts mitochondrial energetics in pulmonary epithelial cells in vivo. Am. J. Physiol..

[32] Sarafian T.A., Kouyoumjian S., Khoshaghideh F., Tashkin D.P., Roth M.D. (2003). Delta 9-tetrahydrocannabinol disrupts mitochondrial function and cell energetics. Am. J. Physiol..

[33] Morimoto S., Tanaka Y., Sasaki K., Tanaka H., Fukamizu T., Shoyama Y., Shoyama Y., Taura F. (2007). Identification and characterization of cannabinoids that induce cell death through mitochondrial permeability transition in Cannabis leaf cells. J. Biol. Chem..

[34] Singh N., Hroudova J., Fisar Z. (2015). Cannabinoid-Induced Changes in the Activity of Electron Transport Chain Complexes of Brain Mitochondria. J. Mol. Neurosci..

[35] Tahir S.K., Zimmerman A.M. (1991). Influence of marihuana on cellular structures and biochemical activities. Pharmacol. Biochem. Behav..

[36] Fisar Z., Singh N., Hroudova J. (2014). Cannabinoid-induced changes in respiration of brain mitochondria. Toxicol. Lett..

[37] Canto C., Menzies K.J., Auwerx J. (2015). NAD(+) Metabolism and the Control of Energy Homeostasis: A Balancing Act between Mitochondria and the Nucleus. Cell Metab..

[38] Scientists Are Baffled by Spatter of Babies Born without Hands or Arms in France, as Investigation Fails to Discover a Cause. https://www.dailymail.co.uk/news/article-7242059/Scientists-baffled-babies-born-without-hands-arms-France-probe-fails-discover-cause.html.

[39] (2018). Agence France-Presse in Paris: France to investigate cause of upper limb defects in babies. The Guardian.

[40] Willsher K. (2018). Baby arm defects prompt nationwide investigation in France. Guardian.

[41] Carlson B.M. (2019). Human Embryology and Developmental Biology.

[42] Eurocat Data: Prevalence Charts and Tables. https://eu-rd-platform.jrc.ec.europa.eu/eurocat/eurocat-data/prevalence_en.

[43] Global Health Observatory. https://www.who.int/data/gho/data/indicators/indicator-details/GHO/total-(recorded-unrecorded)-alcohol-per-capita-(15-)-consumption.

[44] European Monitoring Centre for Drugs and Drug Addiction (EMCDDA): Statistical Bulletin 2021—Prevalence of Drug Use. https://www.emcdda.europa.eu/data/stats2021/gps_en.

[45] The World Bank: Crude Data: Adjusted Net National Income per Capita (Current US$). https://data.worldbank.org/indicator/NY.ADJ.NNTY.PC.CD.

[46] R: A Language and Environment for Statistical Computing. https://cran.r-project.org/.

[47] Wickham H., Averick M., Bryan J., Chang W., McGowan L.D., Francios R., Groelmund G., Hayes A., Henry L., Hester J. (2019). Welcome to the Tidyverse. J. Open Source Softw..

[48] Pebesma E. (2018). Simple Features for R: Standardized Support for Spatial Vector Data. R J..

[49] Viridis: Default Color Maps from ‘matplotlib’. https://CRAN.R-project.org/package=viridis.

[50] Colorplaner: Ggplot2 Extension to Visualize Two Variables per Color Aesthetic through Colorspace Projection. https://github.com/wmurphyrd/colorplaner.

[51] The Nlme Package: Linear and Nonlinear Mixed Effects Models, R Version 3. https://www.researchgate.net/publication/272475067_The_Nlme_Package_Linear_and_Nonlinear_Mixed_Effects_Models_R_Version_3.

[52] Broom.Mixed: Tidying Methods for Mixed Models. http://github.com/bbolker/broom.mixed.

[53] Broom: Convert Statistical Objects into Tidy Tibbles. https://CRAN.R-project.org/package=broom.

[54] Leeper T.J. (2021). Margins: Marginal Effects for Model Objects. R Package Version 0.3.26.

[55] Wright M.N., Ziegler A. (2017). Ranger: A Fast Implementation of Random Forests for High Dimensional Data in C++ and R. J. Stat. Softw..

[56] Greenwell B.M., Boehmke B.C. (2021). Variable Importance Plots—An Introduction to the vip Package. R J..

[57] Package ‘plm’. https://cran.r-project.org/web/packages/plm/plm.pdf.

[58] The spdep Package. https://www.rdocumentation.org/packages/spdep/versions/1.2-7.

[59] Millo G., Piras G. (2012). Splm: Spatial Panel Data Models in R. J. Stat. Softw..

[60] Package ‘splm’. https://cran.r-project.org/web/packages/splm/splm.pdf.

[61] Croissant Y., Millo G. (2019). Panel Data Econometrics with R.

[62] Wal W., Geskus R. (2011). Ipw: An R Package for Inverse Probability Weighting. J. Stat. Softw..

[63] VanderWeele T.J., Ding P. (2017). Sensitivity Analysis in Observational Research: Introducing the E-Value. Ann. Intern. Med..

[64] VanderWeele T.J., Martin J.N., Mathur M.B. (2020). E-values and incidence density sampling. Epidemiology.

[65] VanderWeele T.J., Mathur M.B. (2020). Commentary: Developing best-practice guidelines for the reporting of E-values. Int. J. Epidemiol..

[66] VanderWeele T.J., Ding P., Mathur M. (2019). Technical Considerations in the Use of the E-Value. J. Causal Inference.

[67] Pearl J., Mackaenzie D. (2019). The Book of Why: The New Science of Cause and Effect.

[68] Package ‘Evalue’. https://cran.r-project.org/web/packages/EValue/EValue.pdf.

[69] McKowen J., Woodward D., Yule A.M., DiSalvo M., Rao V., Greenbaum J., Joshi G., Wilens T.E. (2022). Characterizing autistic traits in treatment-seeking young adults with substance use disorders. Am. J. Addict..

[70] Schrott R., Acharya K., Itchon-Ramos N., Hawkey A.B., Pippen E., Mitchell J.T., Kollins S.H., Levin E.D., Murphy S.K. (2019). Cannabis use is associated with potentially heritable widespread changes in autism candidate gene DLGAP2 DNA methylation in sperm. Epigenetics.

[71] Hill A.B. (1965). The Environment and Disease: Association or Causation?. Proc. R. Soc. Med..

[72] Vallee A., Lecarpentier Y., Guillevin R., Vallee J.N. (2017). Effects of cannabidiol interactions with Wnt/beta-catenin pathway and PPARgamma on oxidative stress and neuroinflammation in Alzheimer’s disease. Acta Biochim. Biophys. Sin..

[73] Nallathambi R., Mazuz M., Namdar D., Shik M., Namintzer D., Vinayaka A.C., Ion A., Faigenboim A., Nasser A., Laish I. (2018). Identification of Synergistic Interaction Between Cannabis-Derived Compounds for Cytotoxic Activity in Colorectal Cancer Cell Lines and Colon Polyps That Induces Apoptosis-Related Cell Death and Distinct Gene Expression. Cannabis Cannabinoid Res..

[74] Petko J., Tranchina T., Patel G., Levenson R., Justice-Bitner S. (2018). Identifying novel members of the Wntless interactome through genetic and candidate gene approaches. Brain Res. Bull..

[75] Xian X., Tang L., Wu C., Huang L. (2018). miR-23b-3p and miR-130a-5p affect cell growth, migration and invasion by targeting CB1R via the Wnt/beta-catenin signaling pathway in gastric carcinoma. OncoTargets Ther..

[76] McKenzie M.G., Cobbs L.V., Dummer P.D., Petros T.J., Halford M.M., Stacker S.A., Zou Y., Fishell G.J., Au E. (2019). Non-canonical Wnt Signaling through Ryk Regulates the Generation of Somatostatin- and Parvalbumin-Expressing Cortical Interneurons. Neuron.

[77] Nalli Y., Dar M.S., Bano N., Rasool J.U., Sarkar A.R., Banday J., Bhat A.Q., Rafia B., Vishwakarma R.A., Dar M.J. (2019). Analyzing the role of cannabinoids as modulators of Wnt/beta-catenin signaling pathway for their use in the management of neuropathic pain. Bioorg. Med. Chem. Lett..

[78] Birerdinc A., Jarrar M., Stotish T., Randhawa M., Baranova A. (2013). Manipulating molecular switches in brown adipocytes and their precursors: A therapeutic potential. Prog. Lipid Res..

[79] Richard D., Picard F. (2011). Brown fat biology and thermogenesis. Front. Biosci..

[80] Xu T.R., Yang Y., Ward R., Gao L., Liu Y. (2013). Orexin receptors: Multi-functional therapeutic targets for sleeping disorders, eating disorders, drug addiction, cancers and other physiological disorders. Cell. Signal..

[81] Fraher D., Ellis M.K., Morrison S., McGee S.L., Ward A.C., Walder K., Gibert Y. (2015). Lipid Abundance in Zebrafish Embryos Is Regulated by Complementary Actions of the Endocannabinoid System and Retinoic Acid Pathway. Endocrinology.

[82] Kučukalić S., Ferić Bojić E., Babić R., Avdibegović E., Babić D., Agani F., Jakovljević M., Kučukalić A., Bravo Mehmedbašić A., Šabić Džananović E. (2019). Genetic Susceptibility to Posttraumatic Stress Disorder: Analyses of the Oxytocin Receptor, Retinoic Acid Receptor-Related Orphan Receptor A and Cannabinoid Receptor 1 Genes. Psychiatr. Danub..

[83] (2012). Lee YS, Jeong WI: Retinoic acids and hepatic stellate cells in liver disease. J. Gastroenterol. Hepatol..

[84] Aguado T., Romero E., Monory K., Palazuelos J., Sendtner M., Marsicano G., Lutz B., Guzmán M., Galve-Roperh I. (2007). The CB1 cannabinoid receptor mediates excitotoxicity-induced neural progenitor proliferation and neurogenesis. J. Biol. Chem..

[85] Williams E.J., Walsh F.S., Doherty P. (2003). The FGF receptor uses the endocannabinoid signaling system to couple to an axonal growth response. J. Cell Biol..

[86] Schrott R., Murphy S.K., Modliszewski J.L., King D.E., Hill B., Itchon-Ramos N., Raburn D., Price T., Levin E.D., Vandrey R. (2021). Refraining from use diminishes cannabis-associated epigenetic changes in human sperm. Environ. Epigenet..

[87] GeneCards: GRIA1. https://www.genecards.org/cgi-bin/carddisp.pl?gene=GRIA1&keywords=GRIA1.

[88] GeneCards: GRIK2. https://www.genecards.org/cgi-bin/carddisp.pl?gene=GRIK2&keywords=GRIK2.

[89] GeneCards: GRIN. https://www.genecards.org/cgi-bin/carddisp.pl?gene=GRIN2C&keywords=GRIN2c.

[90] GeneCards: GRID. https://www.genecards.org/cgi-bin/carddisp.pl?gene=GRID2&keywords=GRID2.

[91] GeneCards: GRM3. https://www.genecards.org/cgi-bin/carddisp.pl?gene=GRM3&keywords=GRM3.

[92] GeneCards: GRBR. https://www.genecards.org/cgi-bin/carddisp.pl?gene=GABRE&keywords=GABR.

[93] GeneCards: GRBRA. https://www.genecards.org/cgi-bin/carddisp.pl?gene=GABRA1&keywords=GABRA.

[94] GeneCards: GABRB. https://www.genecards.org/cgi-bin/carddisp.pl?gene=GABRB1&keywords=GABRB.

[95] GeneCards: HTR. https://www.genecards.org/cgi-bin/carddisp.pl?gene=HTR2A&keywords=HTR2A.

[96] GeneCards: DRD. https://www.genecards.org/cgi-bin/carddisp.pl?gene=DRD2&keywords=DRD2.

[97] GeneCards: SLC6A3/DAT. https://www.genecards.org/cgi-bin/carddisp.pl?gene=SLC6A3&keywords=SLC6A3.

[98] GeneCards: ORPM1. https://www.genecards.org/cgi-bin/carddisp.pl?gene=OPRM1&keywords=ORPM1.

[99] GeneCards: OPRD1. https://www.genecards.org/cgi-bin/carddisp.pl?gene=OPRD1&keywords=OPRD1.

[100] GeneCards: OXTR. https://www.genecards.org/cgi-bin/carddisp.pl?gene=OXTR&keywords=OXTR.

[101] GeneCards: NRXN. https://www.genecards.org/cgi-bin/carddisp.pl?gene=NRXN1.

[102] GeneCards: NLGN1. https://www.genecards.org/cgi-bin/carddisp.pl?gene=NLGN1&keywords=NLGN1.

[103] Radyushkin K., Hammerschmidt K., Boretius S., Varoqueaux F., El-Kordi A., Ronnenberg A., Winter D., Frahm J., Fischer J., Brose N. (2009). Neuroligin-3-deficient mice: Model of a monogenic heritable form of autism with an olfactory deficit. Genes Brain Behav..

[104] Sun C., Zhang L., Chen G. (2013). An unexpected role of neuroligin-2 in regulating KCC2 and GABA functional switch. Mol. Brain.

[105] Anderson G.R., Aoto J., Tabuchi K., Foldy C., Covy J., Yee A.X., Wu D., Lee S.J., Chen L., Malenka R.C. (2015). Beta-Neurexins Control Neural Circuits by Regulating Synaptic Endocannabinoid Signaling. Cell.

[106] Hishimoto A., Liu Q.R., Drgon T., Pletnikova O., Walther D., Zhu X.G., Troncoso J.C., Uhl G.R. (2007). Neurexin 3 polymorphisms are associated with alcohol dependence and altered expression of specific isoforms. Hum. Mol. Genet..

[107] Wang H. (2016). Endocannabinoid Mediates Excitatory Synaptic Function of β-Neurexins. Commentary: β-Neurexins Control Neural Circuits by Regulating Synaptic Endocannabinoid Signaling. Front. Neurosci..

[108] Foldy C., Malenka R.C., Sudhof T.C. (2013). Autism-associated neuroligin-3 mutations commonly disrupt tonic endocannabinoid signaling. Neuron.

[109] GeneCards: Down Syndrome Cell Adhesion Molecule. https://www.genecards.org/cgi-bin/carddisp.pl?gene=DSCAM.

[110] Shibata M., Pattabiraman K., Lorente-Galdos B., Andrijevic D., Kim S.K., Kaur N., Muchnik S.K., Xing X., Santpere G., Sousa A.M.M. (2021). Regulation of prefrontal patterning and connectivity by retinoic acid. Nature.

[111] Shibata M., Pattabiraman K., Muchnik S.K., Kaur N., Morozov Y.M., Cheng X., Waxman S.G., Sestan N. (2021). Hominini-specific regulation of CBLN2 increases prefrontal spinogenesis. Nature.

[112] Seigneur E., Südhof T.C. (2017). Cerebellins are differentially expressed in selective subsets of neurons throughout the brain. J. Comp. Neurol..

[113] Cardenas A., Villalba A., de Juan Romero C., Pico E., Kyrousi C., Tzika A.C., Tessier-Lavigne M., Ma L., Drukker M., Cappello S. (2018). Evolution of Cortical Neurogenesis in Amniotes Controlled by Robo Signaling Levels. Cell.

[114] Yeh M.L., Gonda Y., Mommersteeg M.T., Barber M., Ypsilanti A.R., Hanashima C., Parnavelas J.G., Andrews W.D. (2014). Robo1 modulates proliferation and neurogenesis in the developing neocortex. J. Neurosci..

[115] Schmidt E.R.E., Zhao H.T., Park J.M., Dipoppa M., Monsalve-Mercado M.M., Dahan J.B., Rodgers C.C., Lejeune A., Hillman E.M.C., Miller K.D. (2021). A human-specific modifier of cortical connectivity and circuit function. Nature.

[116] Alpar A., Tortoriello G., Calvigioni D., Niphakis M.J., Milenkovic I., Bakker J., Cameron G.A., Hanics J., Morris C.V., Fuzik J. (2014). Endocannabinoids modulate cortical development by configuring Slit2/Robo1 signalling. Nat. Commun..

[117] Lu T., Newton C., Perkins I., Friedman H., Klein T.W. (2006). Cannabinoid treatment suppresses the T-helper cell-polarizing function of mouse dendritic cells stimulated with Legionella pneumophila infection. J. Pharmacol. Exp. Ther..

[118] GeneCards: GLI3. https://www.genecards.org/cgi-bin/carddisp.pl?gene=GLI3&keywords=GLI3.

[119] Reece A.S., Hulse G.K., Preedy V., Patel V. (2022). Cannabinoid Genotoxicity and Congenital Anomalies: A Convergent Synthesis of European and USA Datasets. Cannabis, Cannabinoids and Endocannabinoids.

[120] Reece A.S., Hulse G.K. (2023). Chapter 3: Geospatiotemporal and Causal Inferential Analysis of United States Congenital Anomalies as a Function of Multiple Cannabinoid- and Substance-Exposures: Phenocopying Thalidomide and Hundred Megabase-Scale Genotoxicity. Epidemiology of Cannabis: Genotoxicity and Neurotoxicity, Epigenomics and Aging.

[121] Reece A.S., Hulse G.K. (2022). Effects of Cannabis on Congenital Limb Anomalies in 14 European Nations: A Geospatiotemporal and Causal Inferential Study. Environ. Epigenet..

