# Supplementary material for: European Epidemiological Patterns of Cannabis- and Substance-Related Congenital Neurological Anomalies: Geospatiotemporal and Causal Inferential Study
Source: Int J Environ Res Public Health. 2022 Dec 27;20(1):441. doi: 10.3390/ijerph20010441 (PMC9819725; doi:10.3390/ijerph20010441)

Supplementary Figure Captions

Supplementary Figure SF1.: Panelled scatterplots for log (central nervous system congenital anomaly rates) by substance exposure rates for selected anomalies - 2.

Supplementary Figure SF2.: Panelled scatterplots for log (central nervous system congenital anomaly rates) by exposure to various metrics of cannabis for selected anomalies - 2.

Supplementary Figure SF3.: Sequential map-graphs of log (an- / micro- -ophthalmia rates) across surveyed European nations over time, 2010-2019.

Supplementary Figure SF4.: Sequential map-graphs of log (neural tube defect rates) across surveyed European nations over time, 2010-2019.

Supplementary Figure SF5.: Sequential map-graphs of log (eye anomaly rates) across surveyed European nations over time, 2010-2019.

Supplementary Figure SF6.: Sequential map-graphs of last month cannabis use rates : cannabis THC resin concentrations across surveyed European nations over time, 2010-2019.

Supplementary Figure SF7.: Bivariate colorplaner sequential map-graphs of log (an- / micro-ophthalmia rates) by log of last month cannabis use : cannabis resin THC concentration across surveyed European nations over time, 2010-2019.

Supplementary Figure SF8.: Bivariate colorplaner sequential map-graphs of log (neural tube defect anomaly rates) by log of last month cannabis use : cannabis resin THC concentration across surveyed European nations over time, 2010-2019.

Supplementary Figure SF9: Central nervous system congenital anomalies overall over time stratified by daily cannabis use category. See Methods section for details of categorization.

Supplementary Figure SF10: Central nervous system congenital anomalies over time by anomaly stratified by daily cannabis use category.

Supplementary Figure SF11: International geospatial links used in sparse spatial weights matrix (A) edited and (B) final.

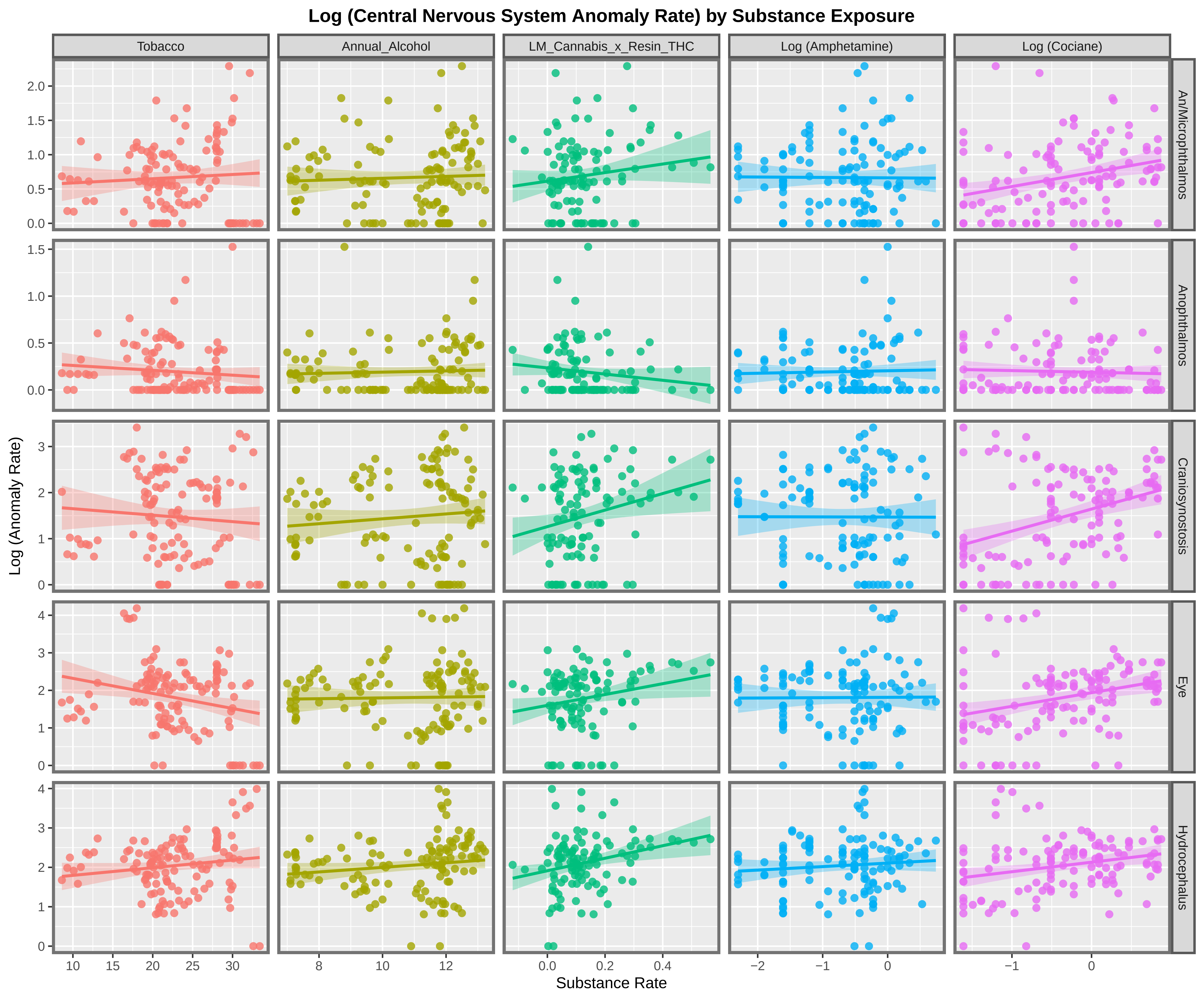

Log (Central Nervous System Anomaly Rate) by Substance Exposure
Tobacco
Annual_Alcohol
LM_Cannabis_x_Resin_THC
Log (Amphetamine)
Log (Cociane)
An/Microphthalmos
Anophthalmos
Craniosynostosis
Eye
Hydrocephalus
Log (Anomaly Rate)
Substance Rate

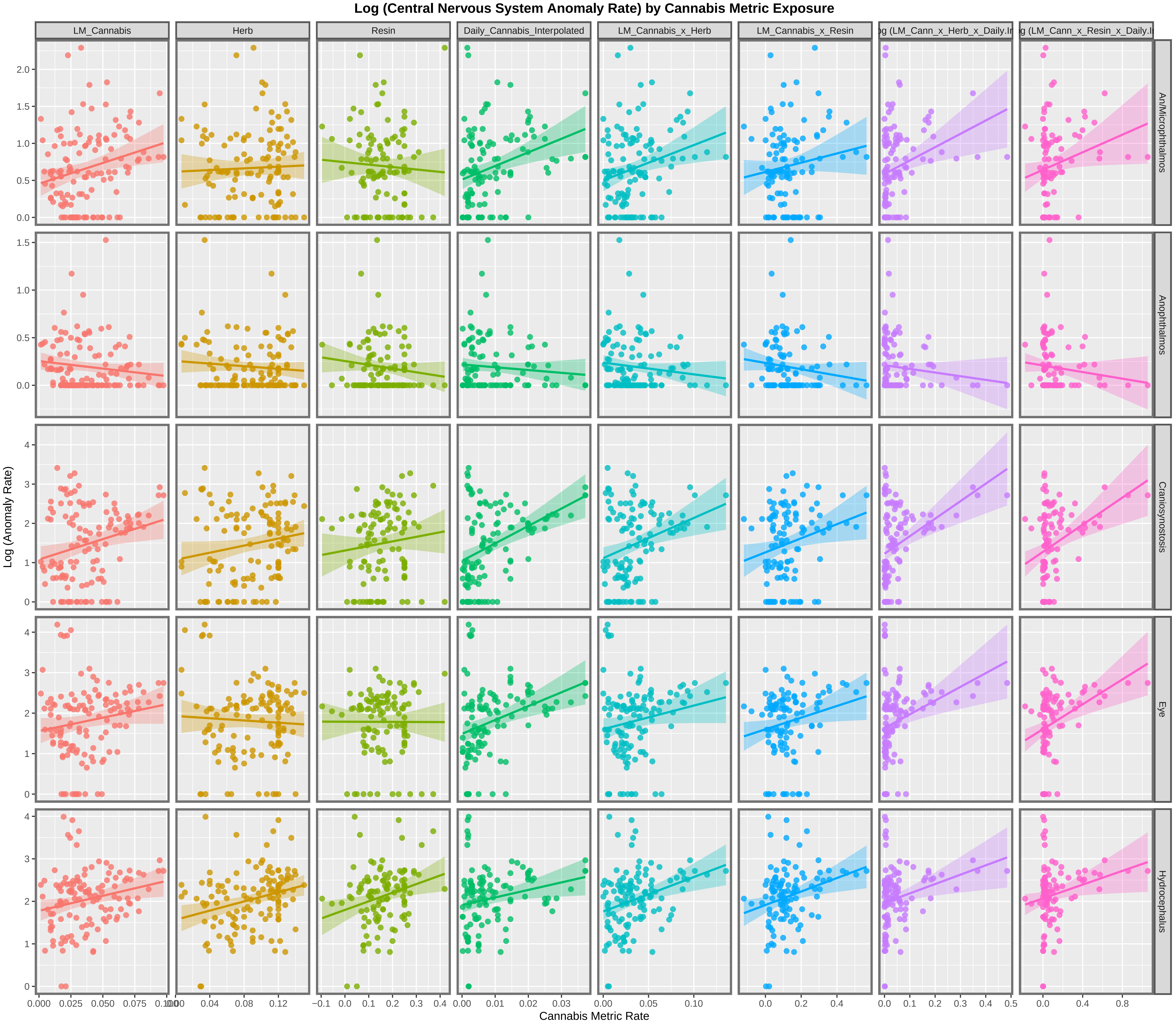

Log (Central Nervous System Anomaly Rate) by Cannabis Metric Exposure
LM_Cannabis
Herb
Resin
Daily_Cannabis_Interpolated
LM_Cannabis_x_Herb
LM_Cannabis_x_Resin
og (LM_Cann_x_Herb_x_Daily.In
g (LM_Cann_x_Resin_x_Daily.I
An/Microphthalmos
Anophthalmos
Craniosynostosis
Eye
Hydrocephalus
Log (Anomaly Rate)
Cannabis Metric Rate

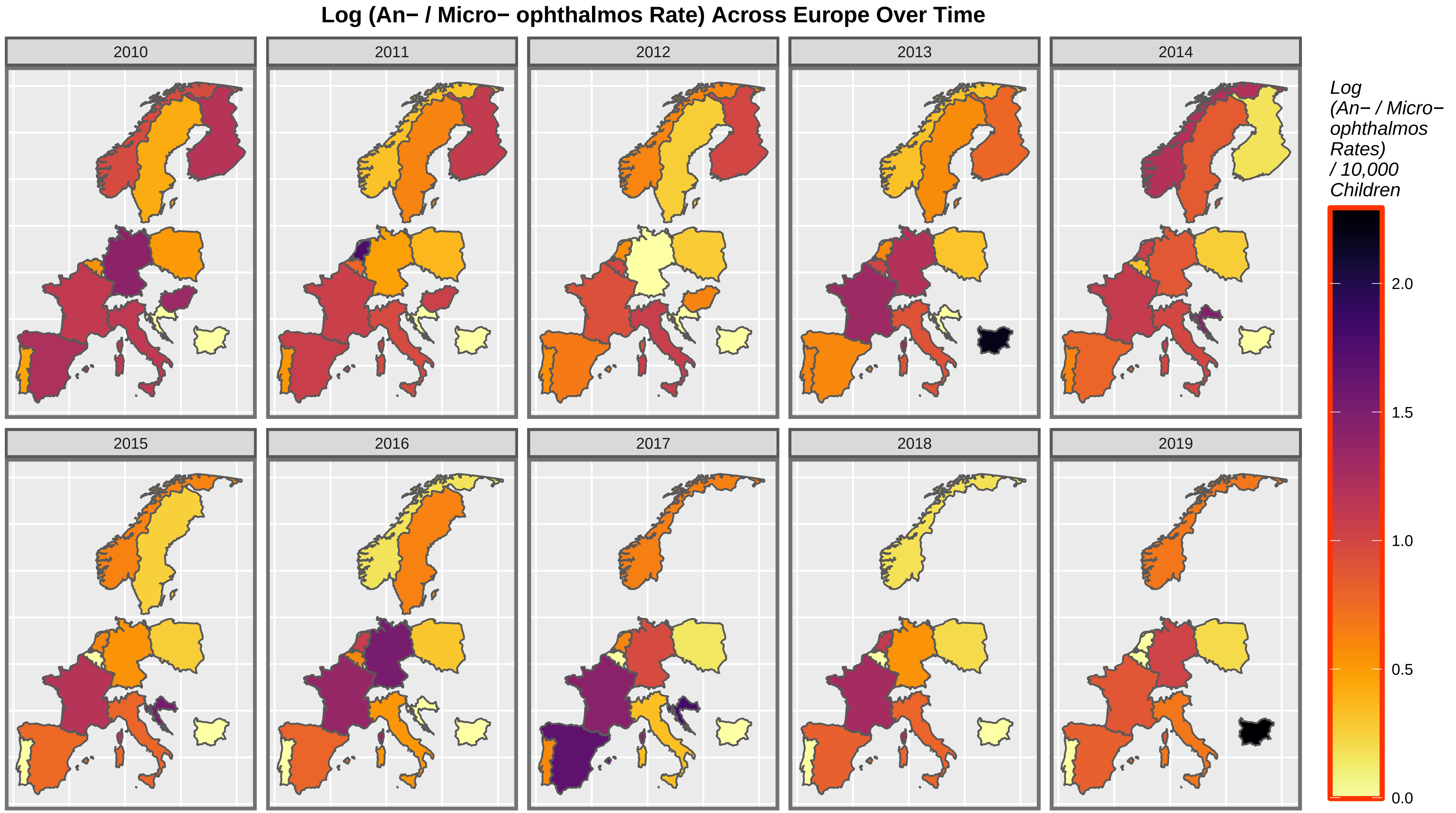

Log (An− / Micro− ophthalmos Rate) Across Europe Over Time
2010
2011
2012
2013
2014
2015
2016
2017
2018
2019
Log
(An− / Micro−
ophthalmos
Rates)
/ 10,000
Children
2.0
1.5
1.0
0.5
0.0

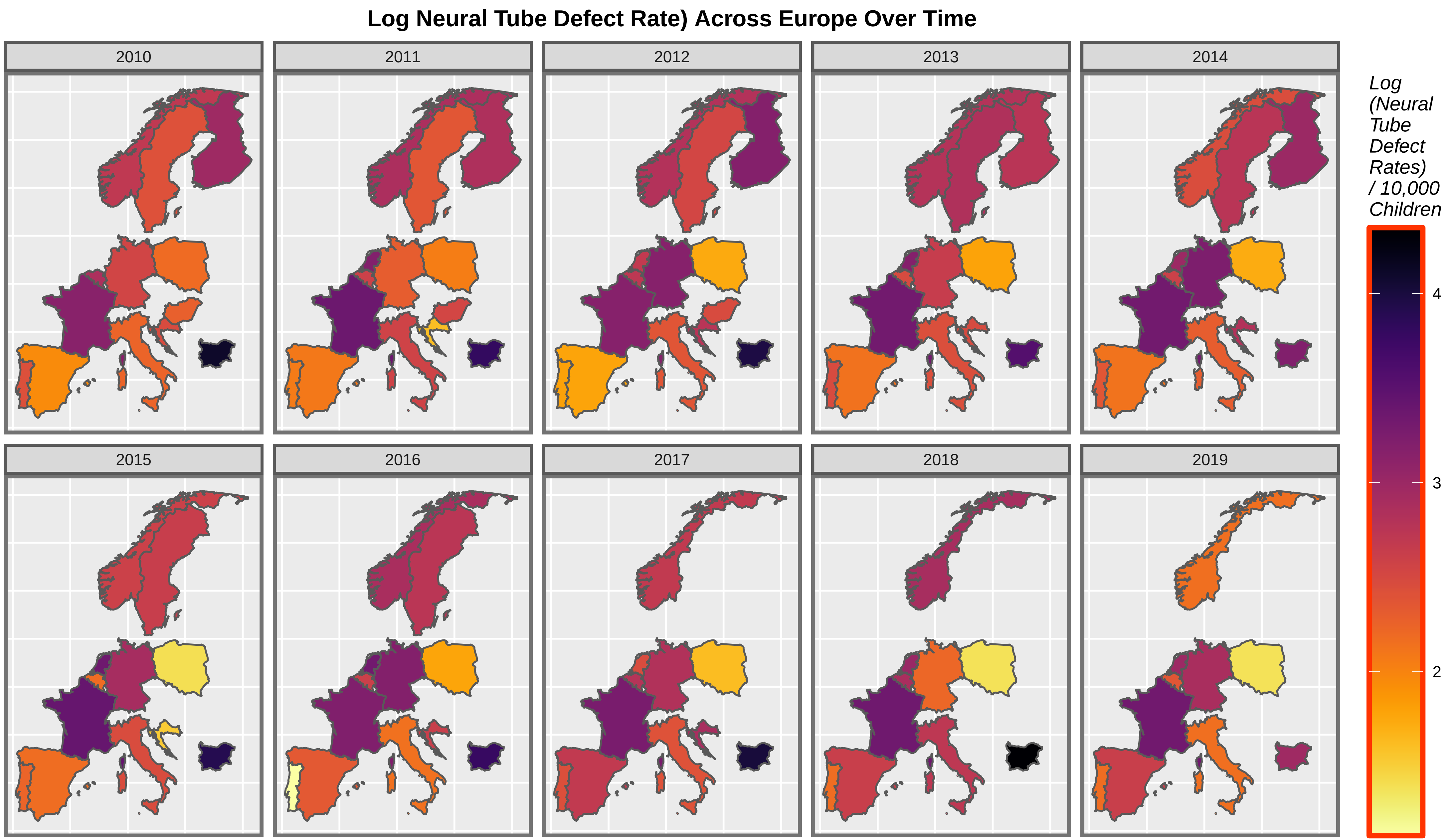

Log Neural Tube Defect Rate) Across Europe Over Time
2010
2011
2012
2013
2014
2015
2016
2017
2018
2019
Log (Neural Tube Defect Rates) / 10,000 Children
4
3
2

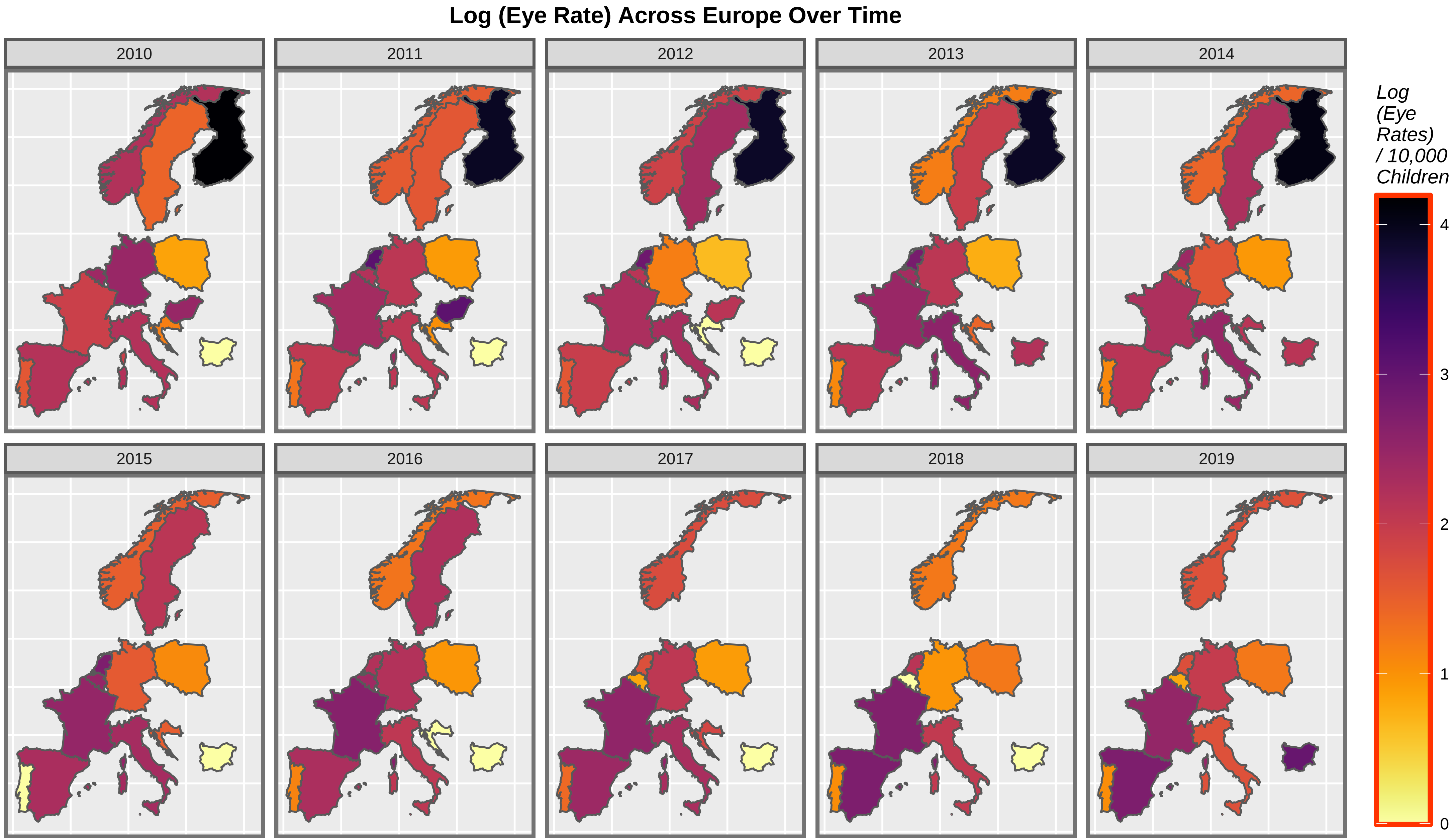

Log (Eye Rate) Across Europe Over Time
2010
2011
2012
2013
2014
2015
2016
2017
2018
2019
Log (Eye Rates) / 10,000 Children
4
3
2
1
0

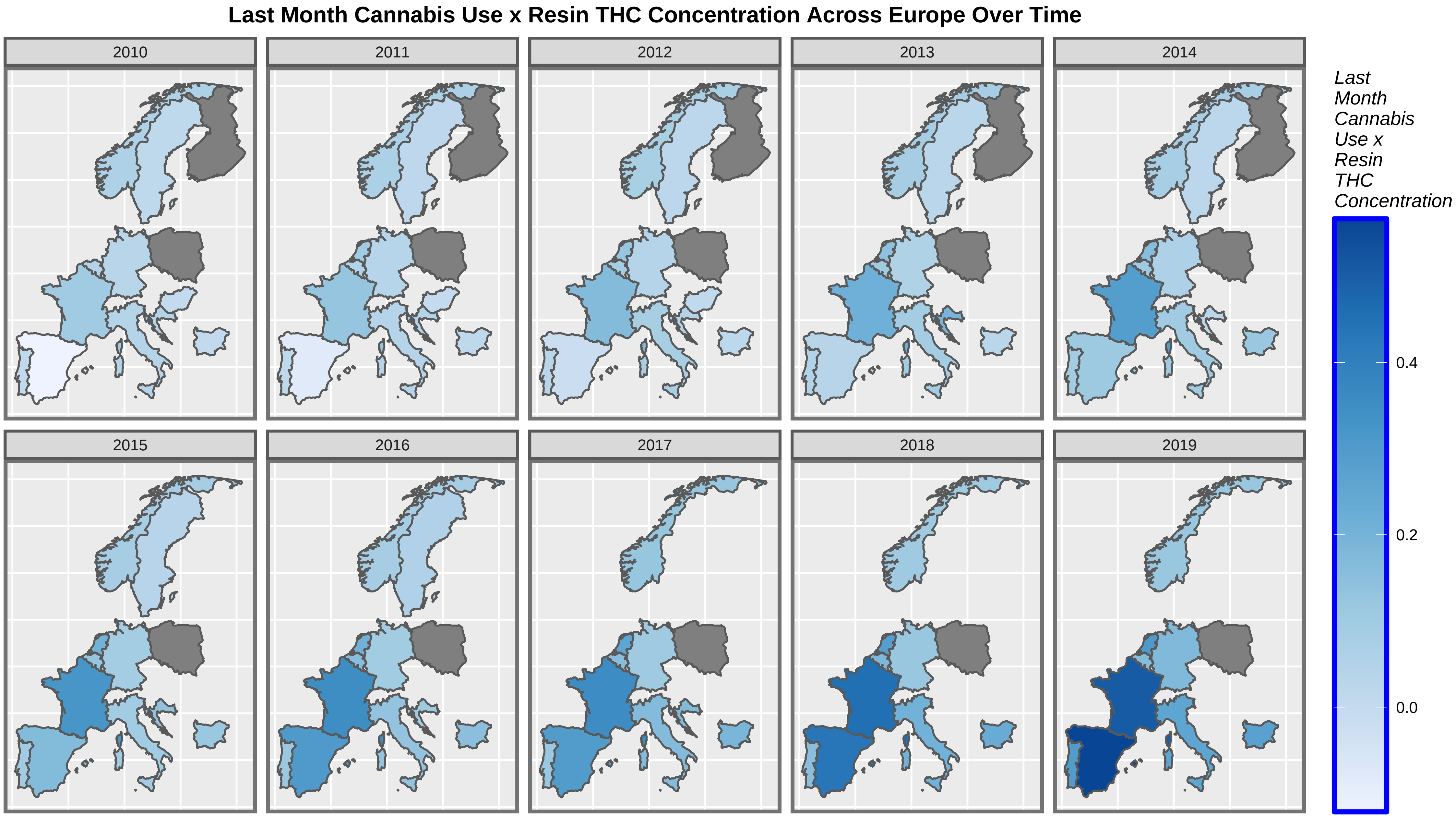
Last Month Cannabis Use x Resin THC Concentration Across Europe Over Time
2010
2011
2012
2013
2014
2015
2016
2017
2018
2019
Last Month Cannabis Use x Resin THC Concentration
0.4
0.2
0.0

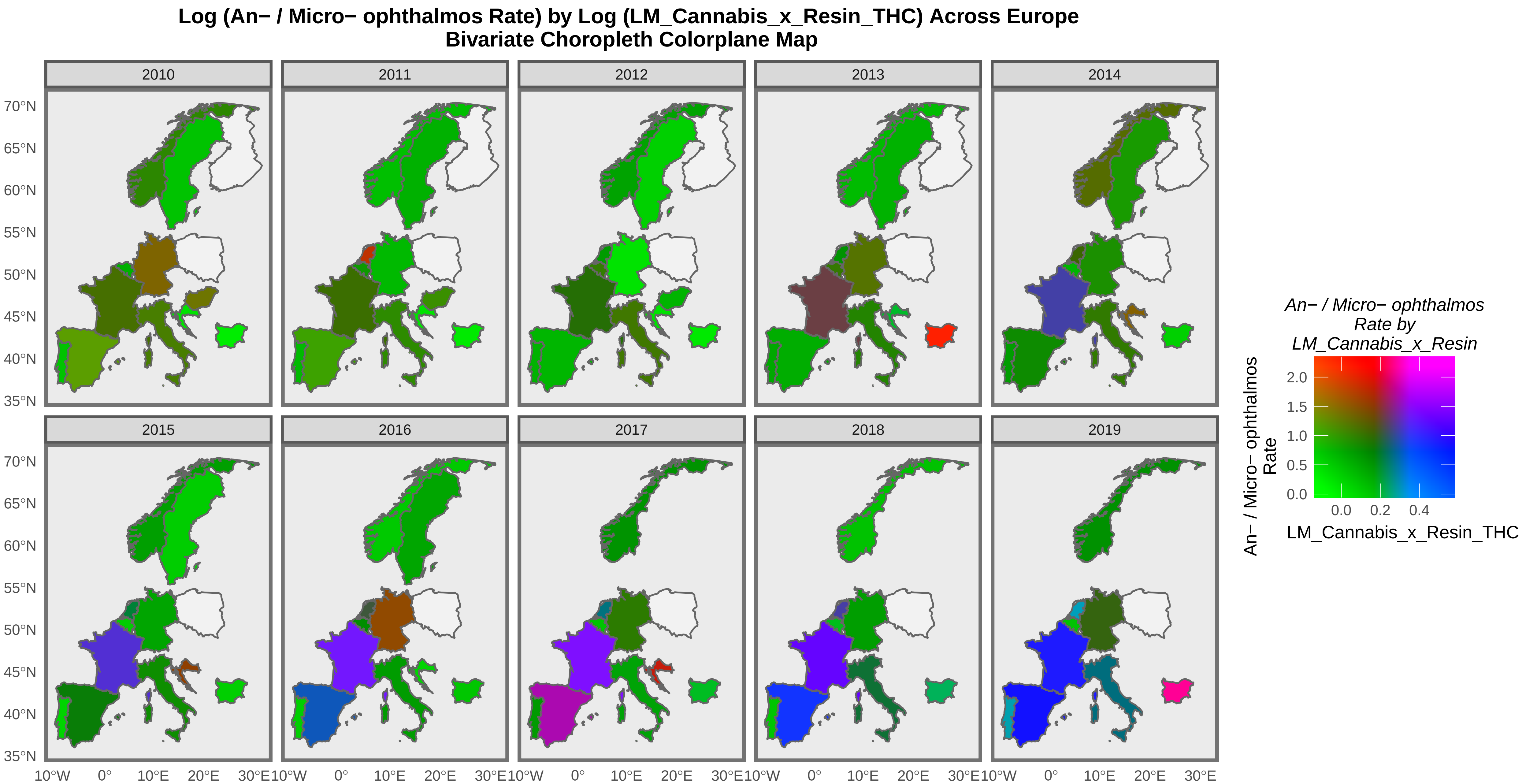
Log (An− / Micro− ophthalmos Rate) by Log (LM_Cannabis_x_Resin_THC) Across Europe
Bivariate Choropleth Colorplane Map
2010
2011
2012
2013
2014
2015
2016
2017
2018
2019
70°N
65°N
60°N
55°N
50°N
45°N
40°N
35°N
10°W
0°
10°E
20°E
30°E
An− / Micro− ophthalmos
Rate by
LM_Cannabis_x_Resin
An− / Micro− ophthalmos
Rate
2.0
1.5
1.0
0.5
0.0
0.0
0.2
0.4
LM_Cannabis_x_Resin_THC

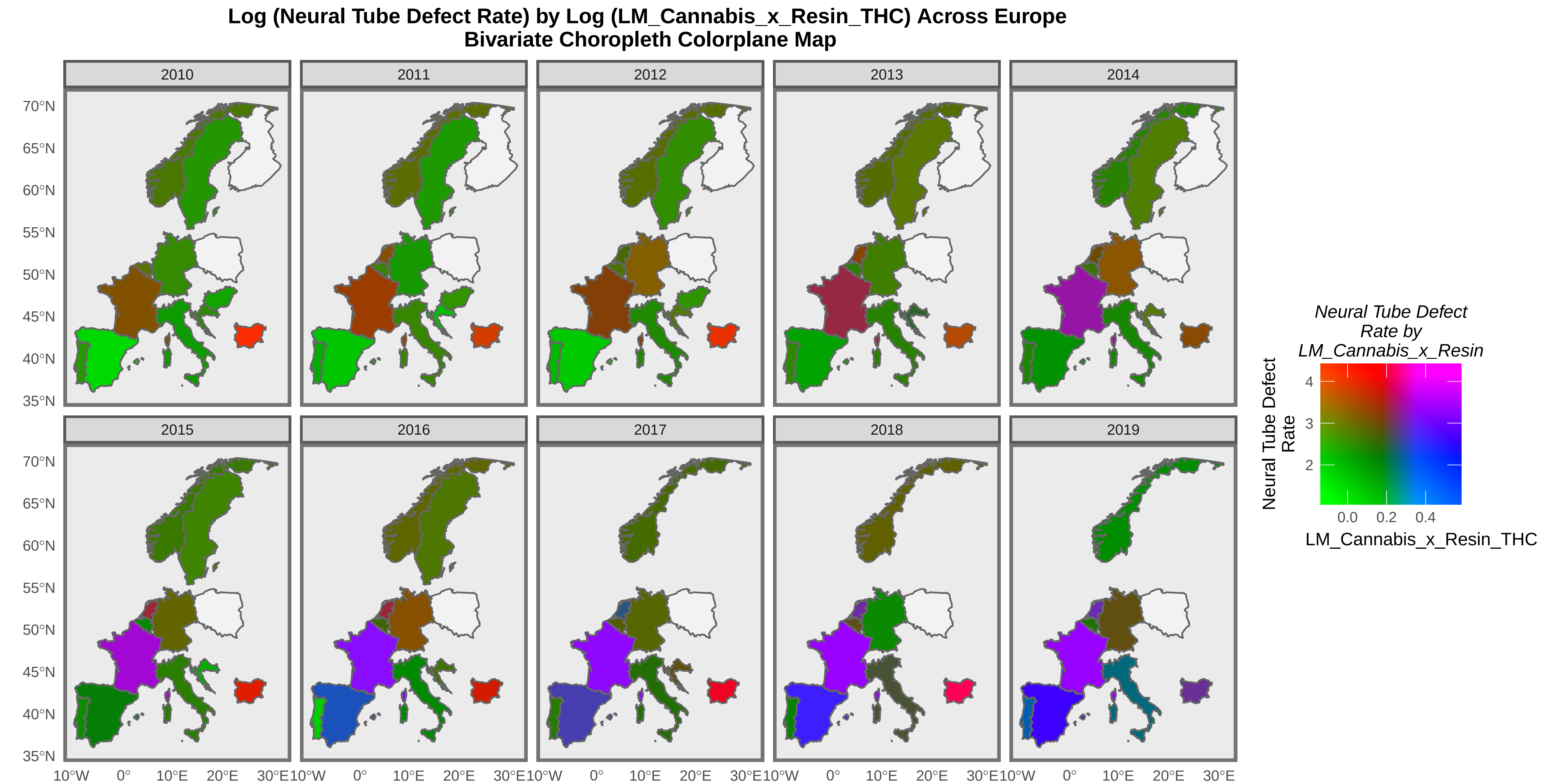

Log (Neural Tube Defect Rate) by Log (LM_Cannabis_x_Resin_THC) Across Europe
Bivariate Choropleth Colorplane Map
2010
2011
2012
2013
2014
2015
2016
2017
2018
2019
70°N
65°N
60°N
55°N
50°N
45°N
40°N
35°N
10°W
0°
10°E
20°E
30°E
Neural Tube Defect Rate by LM_Cannabis_x_Resin
Neural Tube Defect Rate
4
3
2
0.0
0.2
0.4
LM_Cannabis_x_Resin_THC

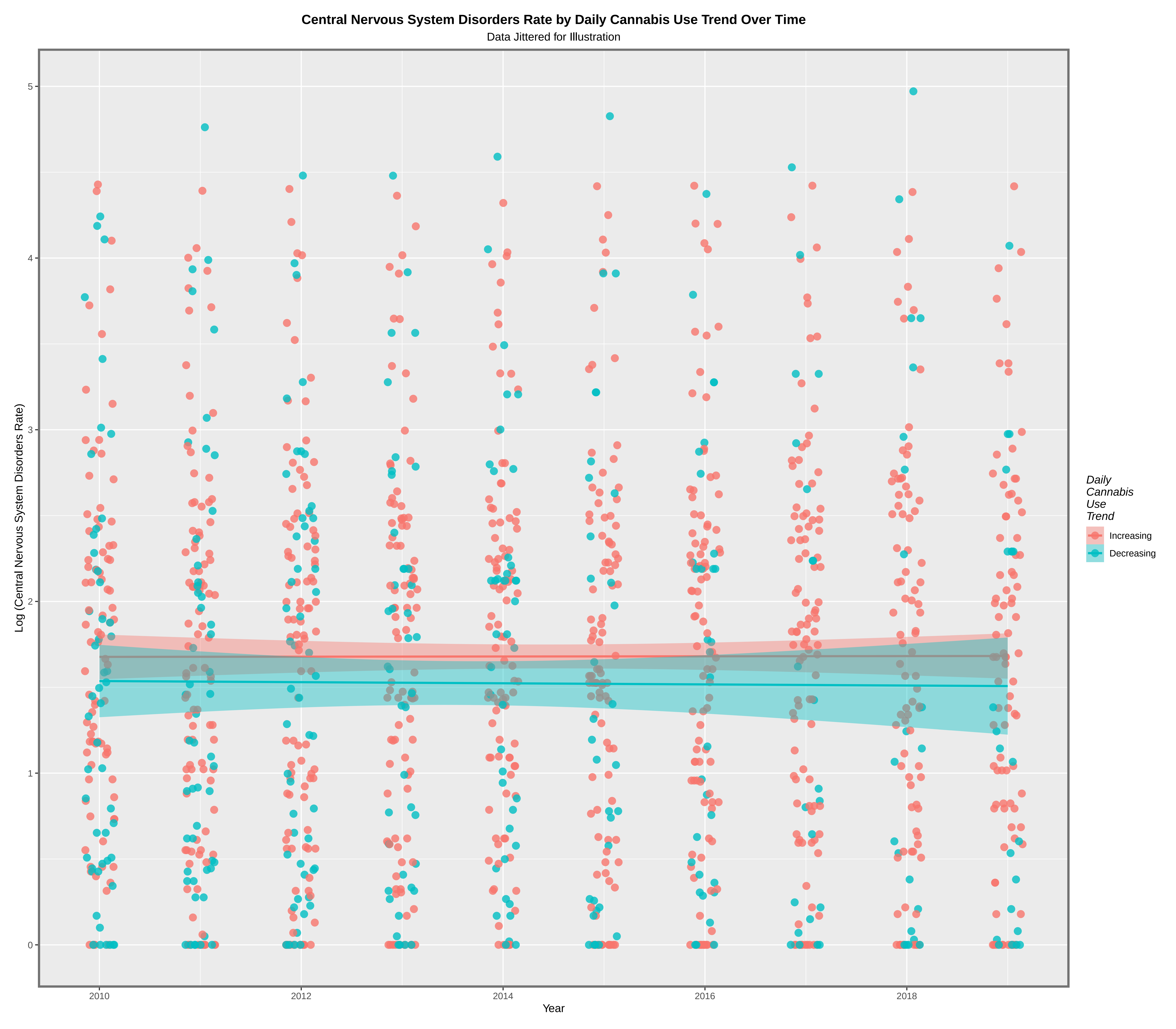

Central Nervous System Disorders Rate by Daily Cannabis Use Trend Over Time
Data Jittered for Illustration
Log (Central Nervous System Disorders Rate)
Year
Daily Cannabis Use Trend
Increasing
Decreasing
0
1
2
3
4
5
2010
2012
2014
2016
2018

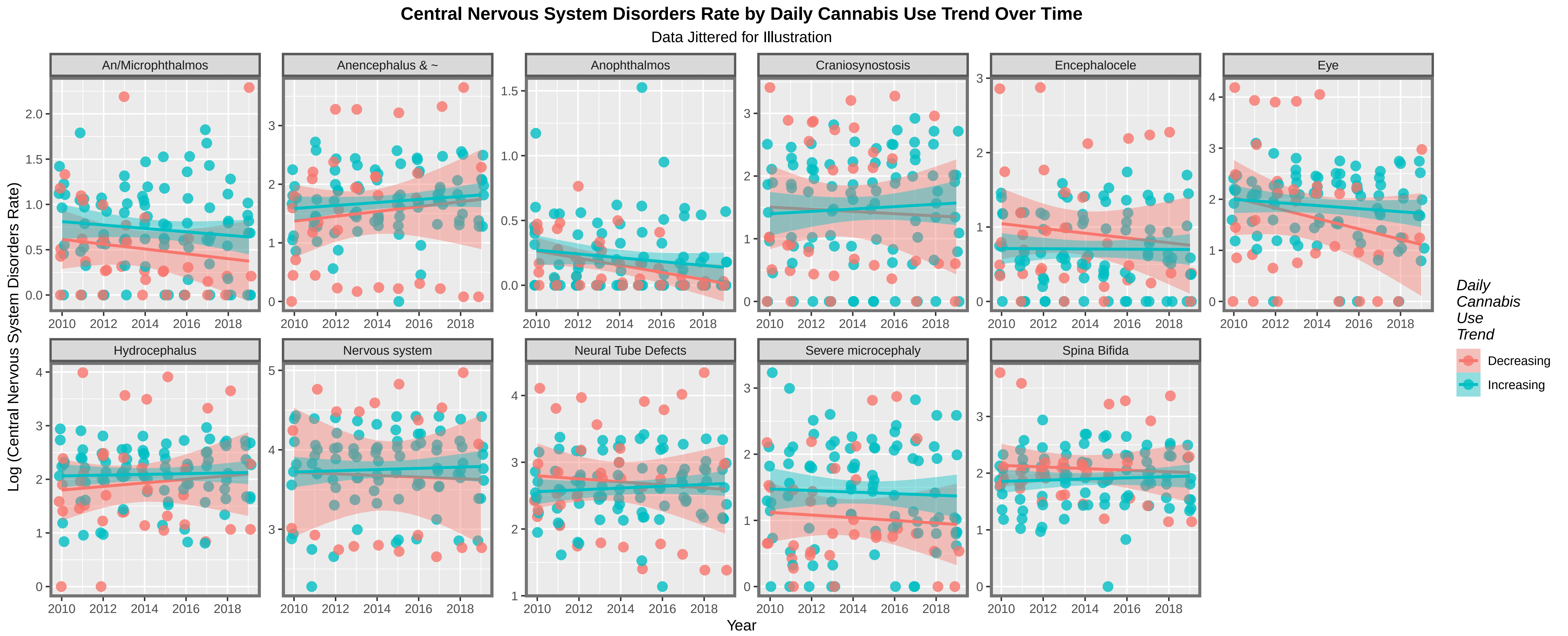
Central Nervous System Disorders Rate by Daily Cannabis Use Trend Over Time
Data Jittered for Illustration
An/Microphthalmos
Anencephalus & ~
Anophthalmos
Craniosynostosis
Encephalocele
Eye
Hydrocephalus
Nervous system
Neural Tube Defects
Severe microcephaly
Spina Bifida
Log (Central Nervous System Disorders Rate)
Year
Daily Cannabis Use Trend
Decreasing
Increasing

Edited (Red) Links (Blue) to European Country Neighbourhood Links for Nervous System Anomalies Dataset

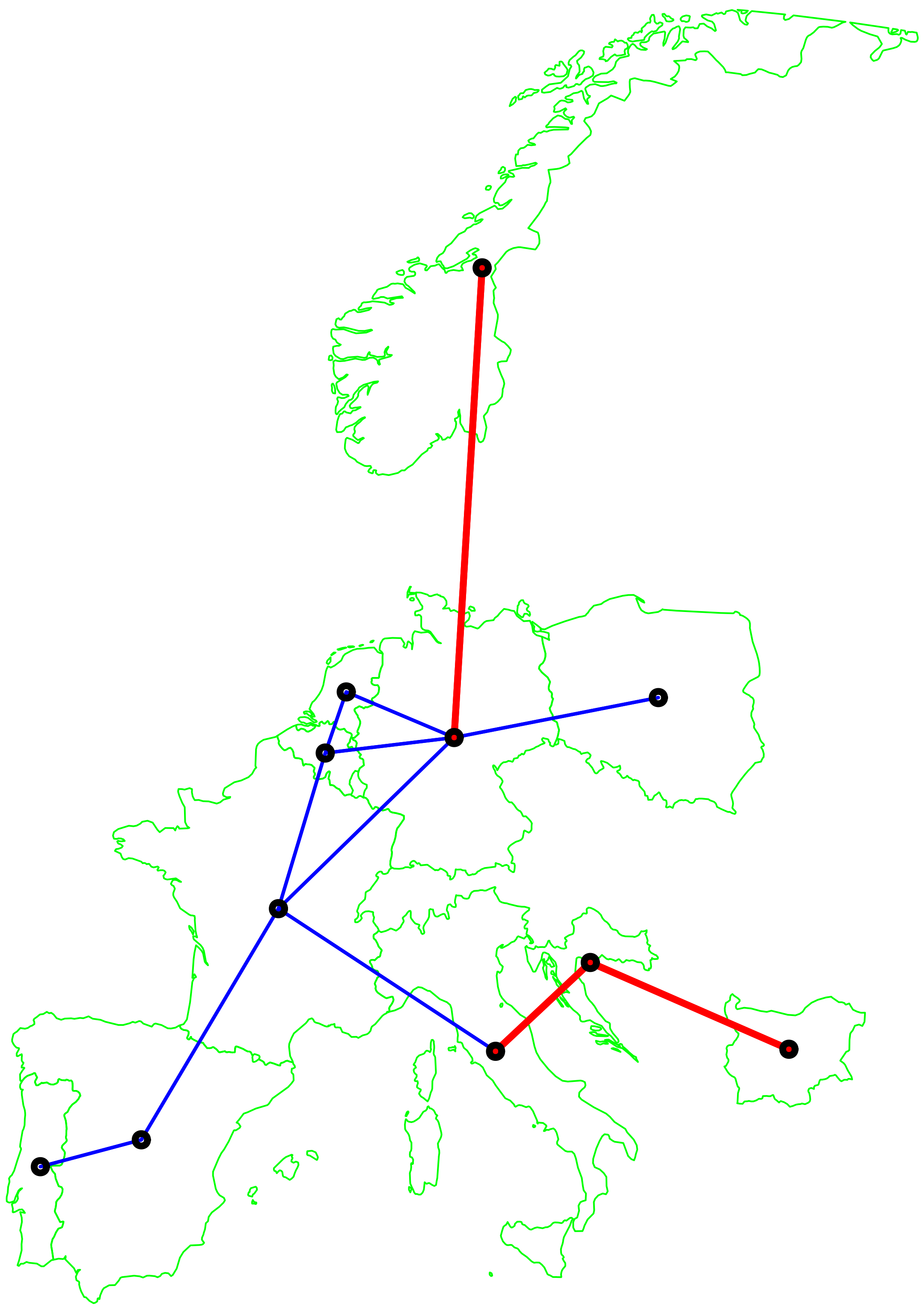

Final Country Neighbourhood Links for European Nervous System Anomalies Dataset

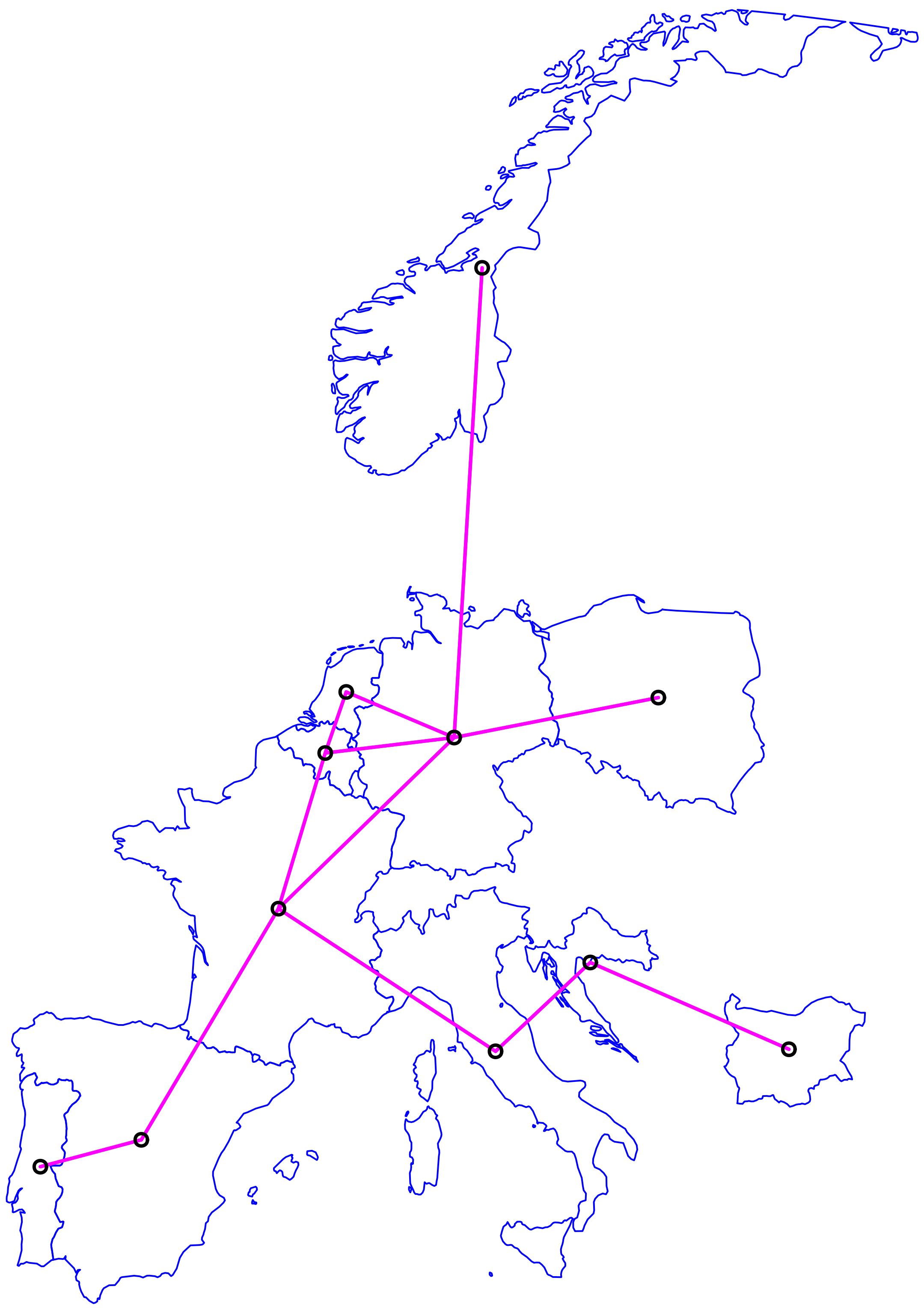

Supplement: Supplementary file 1 [file ijerph-20-00441-s001.zip › Supplementary Figures 1-11 Eur_CNS w Captions.pdf]
